# Gene Expression Profiling of Muscle Stem Cells Identifies Novel Regulators of Postnatal Myogenesis

**DOI:** 10.3389/fcell.2016.00058

**Published:** 2016-06-21

**Authors:** Sonia Alonso-Martin, Anne Rochat, Despoina Mademtzoglou, Jessica Morais, Aurélien de Reyniès, Frédéric Auradé, Ted Hung-Tse Chang, Peter S. Zammit, Frédéric Relaix

**Affiliations:** ^1^Institut Mondor de Recherche Biomédicale, INSERM U955-E10Créteil, France; ^2^Université Paris Est, Faculté de MedecineCréteil, France; ^3^Ecole Nationale Veterinaire d'AlfortMaison Alfort, France; ^4^Programme Cartes d'Identité des Tumeurs, Ligue Nationale Contre le CancerParis, France; ^5^Sorbonne Universités, UPMC Univ Paris 06, INSERM UMRS974, Center for Research in MyologyParis, France; ^6^Randall Division of Cell and Molecular Biophysics, King's College LondonLondon, UK; ^7^Etablissement Français du SangCréteil, France; ^8^APHP, Hopitaux Universitaires Henri Mondor, DHU Pepsy and Centre de Référence des Maladies Neuromusculaires GNMHCréteil, France

**Keywords:** skeletal muscle, myogenesis, satellite cells, ephrins, zinc fingers

## Abstract

Skeletal muscle growth and regeneration require a population of muscle stem cells, the satellite cells, located in close contact to the myofiber. These cells are specified during fetal and early postnatal development in mice from a Pax3/7 population of embryonic progenitor cells. As little is known about the genetic control of their formation and maintenance, we performed a genome-wide chronological expression profile identifying the dynamic transcriptomic changes involved in establishment of muscle stem cells through life, and acquisition of muscle stem cell properties. We have identified multiple genes and pathways associated with satellite cell formation, including set of genes specifically induced (*EphA1, EphA2, EfnA1, EphB1, Zbtb4, Zbtb20*) or inhibited (*EphA3, EphA4, EphA7, EfnA2, EfnA3, EfnA4, EfnA5, EphB2, EphB3, EphB4, EfnB*s, *Zfp354c, Zcchc5, Hmga2*) in adult stem cells. Ephrin receptors and ephrins ligands have been implicated in cell migration and guidance in many tissues including skeletal muscle. Here we show that Ephrin receptors and ephrins ligands are also involved in regulating the adult myogenic program. Strikingly, impairment of EPHB1 function in satellite cells leads to increased differentiation at the expense of self-renewal in isolated myofiber cultures. In addition, we identified new transcription factors, including several zinc finger proteins. ZFP354C and ZCCHC5 decreased self-renewal capacity when overexpressed, whereas ZBTB4 increased it, and ZBTB20 induced myogenic progression. The architectural and transcriptional regulator HMGA2 was involved in satellite cell activation. Together, our study shows that transcriptome profiling coupled with myofiber culture analysis, provides an efficient system to identify and validate candidate genes implicated in establishment/maintenance of muscle stem cells. Furthermore, tour de force transcriptomic profiling provides a wealth of data to inform for future stem cell-based muscle therapies.

## Introduction

During vertebrate development, successive phases of embryonic and fetal myogenesis leads to formation and growth of skeletal muscles (Relaix et al., 2005; Relaix, 2006; Buckingham and Relaix, 2007). Skeletal muscle cells of trunk and limbs in mouse originate from the early somites, which appear at mid-gestation from undifferentiated presomitic mesoderm (Tajbakhsh and Buckingham, 2000). Following several steps of somite maturation, a population of muscle progenitor cells (MPC) that express the paired-box/homeobox transcription factors *Pax3* and *Pax7* emerge in the central region of the developing somite. Similar cell populations are also found in head muscles, though using a different set of transcriptional regulators (Sambasivan et al., 2011). MPC will both self-renew and give rise to all skeletal muscles via activation of a family of four muscle-specific bHLH transcription factors (*Myf5, Mrf4, MyoD*, and *Myog*: myogenin) that induce the myogenic program (Bismuth and Relaix, 2010; Murphy and Kardon, 2011). Around birth, while all MPC maintain the expression of *Pax7, Pax3* expression in only maintained in a subset of muscles (Relaix et al., 2006) (unpublished observations). MPC become in close contact with the muscle fibers in response to different signals, such as those from the Notch pathway (Seale et al., 2000; Zammit et al., 2006a; Tajbakhsh, 2009; Brohl et al., 2012). During establishment of this anatomical niche, emerging satellite cells acquire stem cell-specific characteristics, including self-renewal capacity (Mauro, 1961; Zammit et al., 2006a; Relaix and Marcelle, 2009). During postnatal muscle growth, satellite cells supply myonuclei to maturing myofibers up to approximately postnatal day 21 (P21) before becoming mitotically quiescent (Lepper et al., 2009; White et al., 2010). Adult satellite cells can be activated from their mitotically quiescent state upon injury (Wang and Rudnicki, 2011; Relaix and Zammit, 2012), to proliferate, and co-express *MyoD* and *Pax7*. They then differentiate via activation of *Myog* (and down-regulation of *Pax7*) to repair damaged myofibers, while a subpopulation of satellite cells will self-renew to restore the pool of quiescent satellite cells by down-regulation of *MyoD* (Zammit et al., 2004; Rudnicki et al., 2008; Relaix and Zammit, 2012).

Understanding regulation of myogenic progression from MPCs to muscle stem cells is central to building a comprehensive model of satellite cell function. Many transcriptional networks that control embryogenesis are also important for myogenesis, such as Notch, BMP (bone morphogenetic protein) or WNT proteins (Linker et al., 2003; Ono et al., 2011; Brohl et al., 2012). Furthermore, a balance between extrinsic cues and intracellular signaling pathways, such as IGF, FGF, Notch, and TGF-β, is required to preserve stem cell function (Brack et al., 2008; Kuang et al., 2008; Brack and Rando, 2012; Dumont et al., 2015).

We have characterized the dynamics of skeletal muscle progenitor and postnatal stem cells from embryonic development to adult life, hence deciphering the intrinsic molecular pathways involved in specification and regulation of these muscle stem cells. Using this large microarray analysis of myogenic progenitors and stem cells during development and adult myogenesis, we identified and evaluated several new candidate factors mediating satellite cell specification and function, with a focus here on EPHB1 and several transcriptional regulators, including four zinc finger transcription regulators (Zfp354c, Zcchc5, Zbtb4, and Zbtb20) and HMGA2, co-regulator belonging to the HMGI family of small high-mobility-group (HMG) proteins (Zhou et al., 1995).

### Eph receptors and ephrin ligands

Eph/ephrin signaling has been shown to regulate muscle satellite cell motility and patterning (Stark et al., 2011), but has not been linked with regulation of the myogenic program, except for one recent study implying promotion and maintenance of slow muscle fiber identity postnatally (Stark et al., 2015). Eph receptors belong to a large family of receptor tyrosine kinases (RTK) involved in cell contact-dependent signaling and patterning (Pitulescu and Adams, 2010). EPHs are classified as EphAs or EphBs based on their binding affinity for the ephrin ligands, ephrin-A (EFNA) or ephrin-B (EFNB) (Figures S1A,B). EFNAs are GPI (glycosylphosphatidylinositol)-anchored and lack a cytoplasmic domain while EFNBs are attached to the membrane by a single transmembrane domain containing a short cytoplasmic PDZ-binding motif (Pasquale, 2005). Interestingly, both Eph receptors and ephrin ligands are competent to signal following interaction (forward and reverse signaling, respectively), and both *trans* and *cis* signaling have been described (Arvanitis and Davy, 2008; Pitulescu and Adams, 2010). In addition, Eph/ephrin signaling is often part of a complex signaling network of regulatory pathways, for instance with adhesion molecules, other cell surface receptors or channels and pores (Arvanitis and Davy, 2008).

Eph/ephrin interaction leads to a large set of developmental processes and biological responses, including adhesion and repulsion, increased or reduced motility, cell plasticity, permeability and morphogenesis, and cell fate specification (Palmer and Klein, 2003; Arvanitis and Davy, 2008). Eph/ephrins are also implicated in regulation of stem cell niches and cancer (Genander and Frisen, 2010; Murai and Pasquale, 2010; Pasquale, 2010).

### Zinc finger transcription factors

Zinc finger proteins belong to a large family of transcription regulators subdivided in seven categories. There are about 800 zinc finger transcription factors in the human genome, with a third of those containing a KRAB (Krüppel Associated Box) domain, such as ZFP354C (see below) or related sequences as ZBTB4 or ZBTB20 (Lupo et al., 2013). KRAB is the most widespread family of transcription factors in the human genome, but is also found in yeast (*S. cerevisiae*) and worm (*C. elegans*) (Ganss and Jheon, 2004). The KRAB protein domain is a powerful repression region that acts as a transcriptional repressor, allowing the binding to co-repressor proteins (Urrutia, 2003). KRAB-containing proteins involved in cell proliferation, differentiation, apoptosis, and tumor formation have been described (Urrutia, 2003; Tian et al., 2006; Li et al., 2008).

*Zfp354c* (*Kid3, AJ18*) belongs to the *Kid* family of genes. The corresponding proteins *Kid1, Kid2*, and *Kid3*, share a very similar structure: a KRAB domain and 11–13 C2H2 motifs (Figure S1C), these last zinc finger motifs consisting of two cysteine and two histidine residues bonded tetrahedrally to a Zinc ion (Ganss and Jheon, 2004). ZFP354c has been previously described as abundant in the brain (Watson et al., 2000), but its expression has not been tested in skeletal muscle. Interestingly, KRAB/C2H2 zinc finger protein ZFP354C participates in the BMP signaling pathway (Jheon et al., 2003), a key regulator of skeletal muscle development and stem cell function (Amthor et al., 1998; Wang et al., 2010; Ono et al., 2011; Sartori et al., 2013). Given the important role of BMP signaling in skeletal muscle biology, ZFP354C is a good candidate as possible regulator of myogenesis.

Zinc finger and BTB domain-containing protein 4 (*Zbtb4, KAISO-L1, Znf903*) is a transcriptional repressor of specificity protein (Sp) transcription factors (Sreevalsan and Safe, 2013), that binds methylated DNA to repress transcription (Filion et al., 2006; Weber et al., 2008). Despite its broad distribution, ZBTB4 is particularly expressed in the brain. In addition, examination of publicly available microarray data sets demonstrated an inverse relationship in the prognostic value and expression of ZBTB4 and the histone methyltransferase EZH2 in tumors from breast cancer patients (Yang et al., 2014). Indeed, polycomb group protein EZH2 controls self-renewal and safeguards the transcriptional identity of skeletal muscle stem cells (Juan et al., 2011).

Zinc finger and BTB domain-containing protein 20 (*Zbtb20, DPZF, Hof*, *Zfp288*) is a member of a subfamily of zinc finger proteins containing C2H2 Krüppel-type zinc fingers and BTB/POZ domains (Mitchelmore, 2002). ZBTB20 can function as a transcriptional repressor and plays an essential role in the specification of pyramidal neurons in the developing hippocampus (Nielsen et al., 2007), and promotes astrocytogenesis during neocortical development (Nagao et al., 2016). ZBTB20 is also a regulator of terminal differentiation of hypertrophic chondrocytes (Zhou et al., 2015). This factor has been recently described to be involved in liver regeneration (Weng et al., 2014), and promoting cell proliferation and tumor growth through repression of FOXO1 (Zhao et al., 2014; Kan et al., 2016). *Zbtb20* null mice exhibit severe postnatal growth retardation, metabolic dysfunction and lethality, suggesting that ZBTB20 plays non-redundant roles in multiple organ systems (Sutherland et al., 2009; Cao et al., 2016).

Zinc finger, CCHC domain-containing 5 (*Zcchc5, Mar3, Zhc5*) belongs to the family of the gag-like retrotransposon genes (glycosaminoglycans) exclusively found in mammals, and is considered an ortholog of Ty3/gypsy group. *Zcchc5* is located on the X chromosome, within the dystrophin (*Dmd*) locus (X21.1) in man. The retrotransposition capacity of these genes seems to have been lost, despite retaining an intact reading frame (Brandt et al., 2005). Thus, the retrotransposons of this family are considered as neogenes with new functions, but their impact and regulation is still poorly understood. *Zcchc5* encodes a nuclear protein containing a CX_2_CX_4_HX_4_C DNA-binding motif, also called CCHC domain, allowing DNA binding to regulate transcription. Furthermore, the proteins of the family of genes *Mart*, which includes *Zcchc5*, have been implicated in the control of cell proliferation and apoptosis in cell lines of liver cancer whereas some become up-regulated in regenerating mouse liver (Okabe et al., 2003; Brandt et al., 2005). Interestingly, *Zcchc5* is expressed in skeletal muscles of the limbs (Diez-Roux et al., 2011) (www.eurexpress.org).

### Architectural factor HMGA2 (HMGI-C)

HMGA2, also called HMGI-C, is a transcriptional co-regulator belonging to the HMGI family of small high-mobility-group (HMG) proteins containing AT-hook DNA binding domains (Zhou et al., 1995). HMGI proteins modulate gene expression by altering chromatin architecture and/or by recruiting other proteins to the transcription regulatory complex (Thanos and Maniatis, 1992; Zhou and Chada, 1998; Pfannkuche et al., 2009). *Hmga2* is highly expressed during embryonic development and down-regulated in most adult tissues (Zhou et al., 1995; Pfannkuche et al., 2009; Ashar et al., 2010). HMGA2 plays an important role in maintaining adult stem/progenitor cells, notably in maintaining neural stem/progenitor cells (Nishino et al., 2008). *Hmga2* is also highly expressed in proliferating skeletal myoblasts during myogenesis, modulating satellite cell activation and proliferation both *in vivo* and *in vitro* (Li et al., 2012). *Hmga2* knockout mice exhibit impaired muscle development and reduced myoblast proliferation, while overexpression of *Hmga2* promotes myoblast growth preventing myoblast differentiation (Li et al., 2012). Thus, HMGA2 is a key regulator of satellite cell activation and skeletal muscle development.

## Methods

### Mice

*Pax3*^*GFP*∕+^ mice (Relaix et al., 2005) were used to isolate MPC by fluorescent activated cell sorting (FACS) of the GFP+ cells. *Pax3*^*Cre*∕+^ mutant mice were kindly provided by Jonathan A. Epstein (Engleka et al., 2005). *R26*^*mT*−*mG*^ mice were obtained from The Jackson Laboratory (Stock No: 007576) (Muzumdar et al., 2007). For myofiber cultures C57BL/6J (Janvier®) male mice (8 weeks old) were used. For lineage tracing experiments, *Pax3*^*Cre*∕+^ mice were crossed with *R26*^*mT*−*mG*^ to obtain *Pax3*^*Cre*∕+^*; R26*^*mT*−*mG*^ double mutant mice.

All animals were maintained inside a barrier facility, and all *in vivo* experiments were performed in accordance with the French and European Community guidelines for the care and use of laboratory animals (Project No: 01427.03 approved by MESR and File No: 15-018 from the Ethical Committee of Anses/ENVA/UPEC).

### Fluorescent activated cell sorting

**Figure 1:**
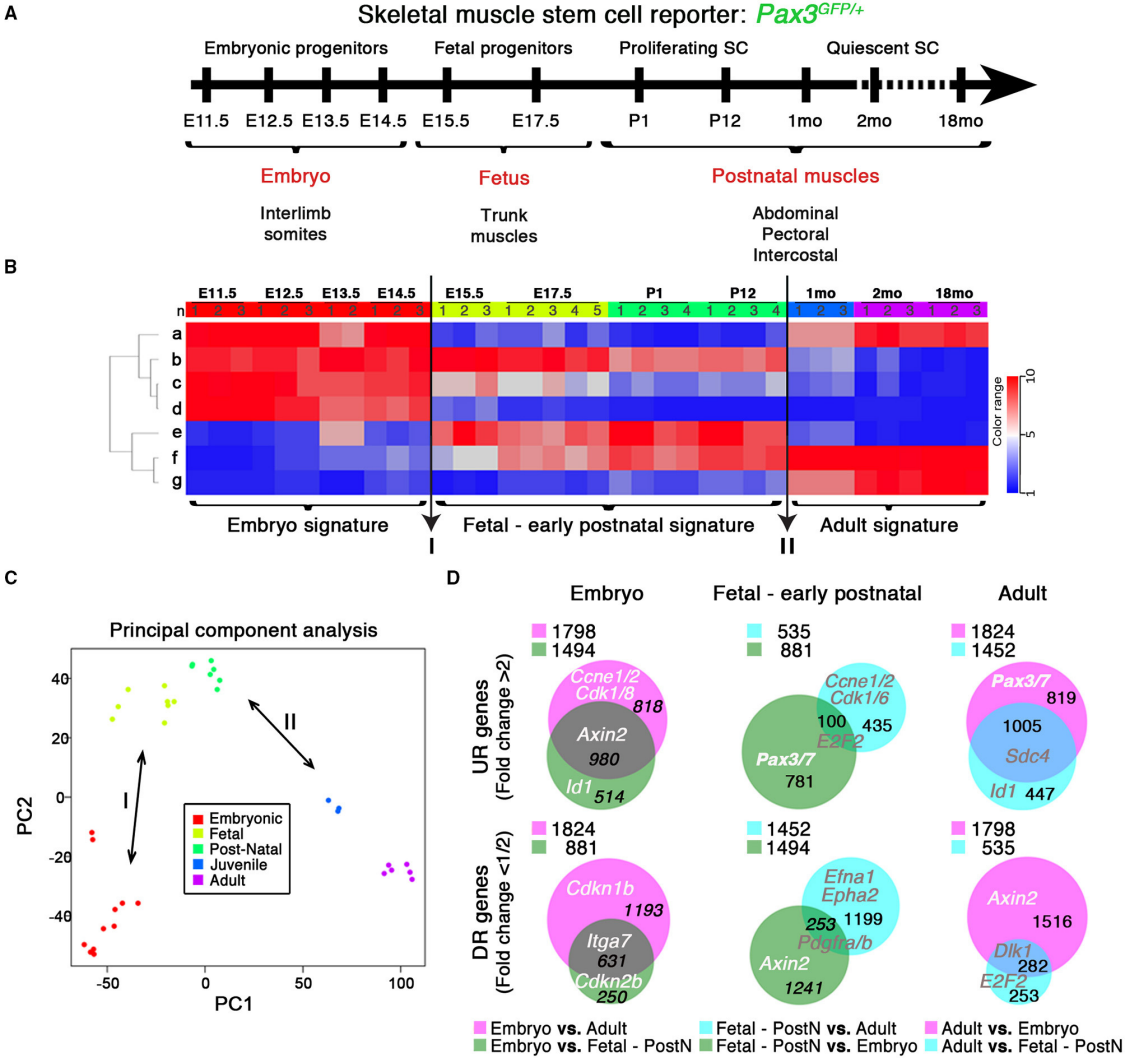
**Transcriptome dynamics from embryonic muscle development to aged mice. (A)** Schematic outline of the experimental procedure illustrating the stages at which *Pax3*^*GFP*∕+^ RNA samples were harvested: E, Embryonic days; P, Postnatal days; mo, age in months. SC, satellite cells. **(B)** Genes from the microarray were organized in seven clusters (“a” to “g”) according to developmental kinetics: with red indicating up-regulated (UR), and blue the down-regulated (DR) transcripts. Transition events (I and II; arrows) are indicated, highlighting the three specific signatures. n, replicate number per stage. **(C)** Principal Component Analysis (PCA) highlights these differences between the three signatures defined by the transitions events in **(B)**. **(D)** Venn diagrams show the interaction of UR (upper panels) or DR (bottom panels) genes from the different comparisons as indicated, illustrating the specific molecular signature for each developmental period. PostN, fetal-to-early postnatal. *p* < 10^−3^ and change fold >2 (UR) or < 1/2 (DR). Gene examples and the number of genes shared between groups are indicated. Legends indicate the total UR or DR genes per comparison.

Trunk muscle samples (intercostal, pectoral and abdominal) were isolated from the trunk as indicated in Figure 1A, at different stages during development and after birth. Muscle were minced and digested in 0.1% Trypsin (Life Technologies®) and 0.1% Collagenase D (Roche®) in DMEM High Glucose without phenol red (Life Technologies®). Digested muscles after filtration were cell-sorted by flow cytometry using a FACS Aria II, using FITC channel to recover the GFP+ cells from *Pax3*^*GFP*∕+^ mice. GFP+ cells were stained using propidium iodide to exclude dead cells (Figure S2A).

### qPCR analysis

RNA from trunk muscles was isolated through the RNeasy Fibrous Tissue kit (Qiagen®). For C2C12, total RNA extraction was performed using the RNeasy mini kit from Qiagen®. Total mRNA content was transcribed into coding DNA (cDNA) according to Transcriptor First Strand cDNA Synthesis kit (Roche®) protocols. Quantitative analyzes were performed using the SYBR-Green kit (Roche®). qPCR was performed on biological duplicates (by sorting two different embryo series) with technical duplicates. The results obtained were analyzed by calculating the 2^∧^-ΔCt. *Hprt1* was used as reference gene.

Oligonucleotides of the following genes were selected, tested and verified according to their efficiencies and specificities:

**Table:** 

*EphB1*:	FWD 5′ - CCGTGGATGACTGGCTAAGT - 3′
	REV 5′ - TACCGATGGTGACTGGTTCA - 3′
*Zbtb4*:	FWD 5′ - CGCTTCTCCATGTTGGCTAT - 3′
	REV 5′ - GTGAGCAGGGAACTTGGTGT - 3′
*Zbtb20*:	FWD 5′ - AATGCGAAAGGGAAGCAGTA - 3′
	REV 5′ - ACAGGACCCGTGGAGTAATG - 3′

### RNA preparation, cDNA synthesis, and microarray hybridization

Microarray processing was performed by PartnerChip (Evry, France), according to NuGEN (http://www.nugen.com/products/microarray-qpcr#cdna-generation) and Affymetrix (http://www.affymetrix.com/support/technical/manuals.affx) protocols. Briefly, total RNA from FACS-sorted trunk muscle GFP+ cells was extracted from independent experiments according to the RNeasy® Micro Kit (QIAGEN) RNA extraction protocol. RNA samples were cleaned using Qiagen RNAeasy mini-columns and their quality assessed by spectrophotometry (Nanodrop ND-1000). Total RNA was analyzed on Agilent microarrays (Bioanalyzer, 2100) to assess integrity of ribosomal RNA (28S and 18S peaks). Synthesis and amplification of cDNA was performed following NuGEN Ovation Pico WTA System protocol, and 100 ng of total RNA were used for first strand cDNA synthesis. Second strand was synthesized following the Ribo-SPIA technology developed by NUGEN. Five micro gram of single-stranded cDNA was fragmented and a biotin-labeled nucleotide was attached to the 3′ end of each fragment (Encore Biotin Module, NuGEN). High-density oligonucleotide arrays containing 45,000 sets of oligonucleotide probes (25 m) that cover all 30,000 genes encoded by the murine genome (Affymetrix Mouse Genome 430 2.0 Arrays, Ref 900495) were used for gene expression detection. Hybridization during 16 h at 45°C in a rotary oven (Affymetrix), washing and staining (GeneChip® Fluidics Station 450) and scanning (GeneChip Scanner 3000) were carried out according to NuGEN and Affymetrix protocols. Expression Console software (Affymetrix) was used for image analysis and to determine probe signal levels. The quality and statistical analysis of the data were finally made using the GeneSpring GX11 analysis software (Agilent Technologies).

### Expression microarray analysis

#### Pre-treatment

Expression profiles of 36 samples (Pax3GFP+ cells at different stages during development and after birth) were obtained using Affymetrix Mouse Genome 430 2.0 Arrays. Expression profiles were normalized in batch using RMA algorithm (affy R package) yielding a (probe sets, samples) matrix. As the 36 samples were obtained by merging two series including 15 and 21 samples, Combat algorithm (Johnson WE—Biostatistics—2007) was used to normalize the corresponding batch effect. Expression profiles were aggregated by Gene Symbol (mean across probe sets) using Affymetrix csv annotation file (na32 version).

#### Unsupervised analysis

The gene expression matrix (GEO Series GSE63860) was then row-mean-centered. The resulting matrix was used for unsupervised classification of the genes. Genes (*n* = 21678) were partitioned in ten clusters using the kmeans classification algorithm. The biggest cluster (*n* = 8896) contained genes showing almost no variation across all samples: it was eliminated from further analysis. Three clusters were found to be highly correlated (centroids correlation >0.95) and were merged in a unique gene cluster (cluster g, Figure 1B). We thus remained with seven clusters. For each sample, the mean expression of all the genes of each cluster was calculated, yielding a (seven clusters, 36 samples)-matrix shown in Figure 1B.

#### Supervised analysis

Moderate *T*-tests (as implemented in limma R package) were used to identify differentially expressed genes.

#### Pathways analysis

To analyze the pathway enrichment, hypergeometric tests were used, taking as “pathways” the terms (and related murine genes) from the Gene Ontology (GO) (http://www.geneontology.org) and the murine KEGG pathways (www.genome.jp/kegg). Pathways enrichment in the seven gene clusters: in each of the seven gene clusters, the pathway analysis was performed using (i) all the genes included in the cluster, (ii) genes selected based on their coefficient of variation and median-absolute-deviation (different thresholds were used): the minimal (hypergeometric test) *p*-value obtained from these different (sub-) lists was retained. Pathways enrichment analysis of differentially expressed genes: given a comparison between two groups of samples, yielding a *p*-value and a fold change for each gene, several lists of differentially expressed genes were selected^*^ and the minimal (hypergeometric test) *p*-value obtained from these different lists was retained. (^*^) Lists of differentially expressed genes: genes yielding a (moderate *T*-test) *p* > 1e-5 were removed from the analysis; remaining genes were ordered based on the fold change; the n genes with highest (respectively lowest) fold change were selected as a separate list; the n/2 genes with highest fold change and the n/2 genes with lowest fold change were merged in another list; this operation was performed for several values of n (200, 300, 400, 500, 750, and 1000). Principal component analysis (PCA) of the expression profiles was performed using R software. Venn diagrams and pathway interaction schemes were generated applying BioVenn (http://www.cmbi.ru.nl/cdd/biovenn/) and GOrilla—REViGO (http://cbl-gorilla.cs.technion.ac.il/) software packages, respectively. Pathway analysis was completed employing DAVID Bioinformatics Resources 6.7 (http://david.abcc.ncifcrf.gov/).

#### Comparative analysis of microarray data with published available datasets

Data normalization was performed with frozenRMA and corrected for batch effect using Combat algorithm (Johnson WE—Biostatistics—2007). Combined data series were the 36 samples from our study and those from published datasets GSE50821 (Sinha et al., 2014) and GSE47177 (Liu et al., 2013). The three Affymetrix series were used to compared adult vs. old expression profiles (supervised meta-analysis young[2 months] vs. old[> = 18 months]). This analysis showed that the combination of the three sets found 32% significantly deregulated genes and in the same direction of deregulation (32% = proportion of the combined test under H1 = Test Stouffer).

#### Accession numbers

The complete microarray data set, including the RMA data used to produce intensity maps, have been deposited in NCBI's Gene Expression Omnibus, and are accessible through GEO Series accession number GSE63860 (http://www.ncbi.nlm.nih.gov/geo/query/acc.cgi?acc=GSE63860).

### Cloning (GMO project no: 371)

To target activated satellite cells and not myofibers in our *ex vivo* assays, a replication-deficient retrovirus, MIGR (*pMSCV-IRES-eGFP*), has been used to transduce proliferating cells and overexpress either dominant negative (DN) *EphB1, Zbtb4*, and *Zbtb20* or full-length cDNA for *Zfp354c, Zcchc5, Zbtb4, Zbtb20*, and *Hmga2* (Pear et al., 1998; Zammit et al., 2006b). The virus is composed, besides the 5′ and 3′ LTR of the MSCV virus, the latter being mutated to prevent replication, and the phi integrase, of a multicloning site followed by an IRES-eGFP sequence to track infected cells by fluorescence. This tracking cassette was later modified into MISSINCK by substituting eGFP with an insulin signal sequence-Cyan Fluorescent Protein (CFP)-KDEL sequence in order to restrict fluorescent tracker expression to the endoplasmic reticulum and Golgi.

### Isolated myofiber cultures

Culture of single fibers was performed according to previously described strategies (Moyle and Zammit, 2014). Briefly, dissected EDL muscles were digested in a filtered solution of 0.2% collagenase (SIGMA-ALDRICH®) in DMEM High Glucose/1% L-Glutamine/1% Penicillin/Streptomycin (Life Technologies®) (isolation medium). After 2 h of connective tissue digestion, EDLs were mechanically dissociated fiber by fiber. Quiescent satellite cells on the isolated myofibers were activated by a solution of 10% horse serum/0.5% chicken embryo extract in filtered isolation medium. Contracted fibers were removed.

### Retrovirus production and myofiber infection

Retroviral particles (see *Cloning*) were produced in HEK293T cells by transfection using FuGENE® with a helper virus, which contains the necessary elements to obtain the correct encapsidation and active retrovirus (*phi integrase, gag, pol* and *env* (VSV-g) genes). We collected the supernatants after transfection at T = 72 h and T = 84 h, which displayed the highest retroviral particle titers.

After 24 h of activation, myofiber-attached satellite cells were infected with the retroviral particles diluted 1/10. 48 h afterwards (T = 72 h), fibers were fixed to proceed with immunofluorescence analysis.

### C2C12 cell culture for muscle differentiation and infection

Myogenic differentiation was induced according to previously reported protocols (McMahon et al., 1994). Murine C2C12 cells were cultured in 10% fetal bovine serum (Bio West®) in High Glucose DMEM (Life Technologies®) for proliferation assay (GM). Differentiation was induced by switching into medium supplemented with 2% horse serum (Promega®) in High Glucose DMEM (DM), generating multinucleated myotubes surrounded by mononuclear reserve cells.

For retroviral infection, 10^4^ C2C12 cells were plated in GM and incubated with undiluted retroviral supernatant containing 4 μg/mL polybrene (SIGMA-ALDRICH®) for 3–4 h. Retroviral medium was then removed, and the cells washed and incubated in either proliferation (for PH3, KI67, EdU, and MYOD analysis) or differentiation (for MYOG analysis) medium.

### Immunostaining

#### Satellite cells on myofibers and cryosections

Myofibers were fixed in 4% paraformaldehyde for 10 min, treated with 0.5% triton and blocked in 10% goat serum/10% swine serum (Moyle and Zammit, 2014). The following antibodies were used: EPHB1 (Rabbit Abcam® ab5414) 1/100, PAX7 monoclonal (DSHB®, PAX7-c) 1/50, MYOD monoclonal (DAKO®, clone 5.8A, M3512) 1/60, MYOD (Rabbit Santa Cruz Biotech®, sc-760) 1/50, MYOG monoclonal (DSHB®, F5D) 1/50, CD-31 (PECAM-1) (Rat BD Pharmigen®, 550274) 1/500, and GFP (Rabbit Life Technologies®) 1/500, or (Chicken Abcam® ab13970) 1/200. Secondary antibodies employed to reveal the staining were Alexa 594 goat anti-mouse IgG (H+L), Alexa 488 goat anti-rabbit IgG (H+L) (Life Technologies®), and DyLight-405 donkey anti-chicken IgY (IgG) (H+L), and Cy5-Goat Anti-Rabbit IgG (H+L) (Jackson ImmunoResearch®). Nuclei were counterstained with DAPI.

### C2C12 cultured myoblasts

The following antibodies were used: MYOD, MYOG, and GFP (as above), PH3 (Rabbit Merck-Millipore®, 06-570) 1/50, Ki67 (BD Pharmigen®, 556003) 1/150, HA (Rabbit Sigma-Aldrich®, H6908) 1/400, and GFP monoclonal (Sigma®) 1/50. Secondary antibodies included Alexa 488 goat anti-mouse IgG (H+L), Alexa 594 goat anti-mouse IgG (H+L), Alexa 488 goat anti-rabbit IgG (H+L), Alexa 594 goat anti-rabbit IgG (H+L) (Life Technologies®). EdU reaction was performed with Click-iT® EdU Alexa Fluor® 647 Imaging Kit (ThermoFisher Scientific®). Nuclei were counterstained with DAPI.

### Imaging and statistics

Analysis was carried out using a Leica TCS SPE confocal microscope. Images were processed with either Adobe Photoshop CS5 software (Adobe Systems) or ImageJ (version 1.47v; National Institutes of Health, USA, http://imagej.nih.gov/ij).

Infected satellite cells in myofiber cultures were directly counted under a Leica fluorescent microscope at 40x and 100x magnification.

Mean ± standard error (SEM) was given. The single (^*^), double (^**^) and triple (^***^) asterisks represent *p*-values *p* < 0.05, *p* < 0.01, and *p* < 0.001 respectively by Student's unpaired *t*-test or Mann–Whitney *U*-test. All experiments have been performed on at least three independent experiments for each condition.

Supplementary Movies were performed using a DSD2 Workstation with Imaris software (ANDOR).

## Results

### Expression dynamics of skeletal muscle stem cells

*Pax3* is expressed in fetal progenitors and satellite cells of trunk hypaxial muscles (Relaix et al., 2005; Relaix, 2006; Calhabeu et al., 2013). We used a Pax3 reporter mouse to perform a chronological global profiling in embryonic, fetal and postnatal MPC and satellite cells expressing Pax3 (Figure 1A; Relaix et al., 2005).

Prospective isolation of Pax3-GFP myogenic progenitors and stem cells was performed as previously described (Figure S2A; Montarras et al., 2005; Lagha et al., 2010), taking advantage of the GFP coding sequence targeting one allele of *Pax3* (Relaix et al., 2005). *Pax3* is expressed in muscle progenitors but also in early migrating neural crest cells (Epstein et al., 1993). Neural crest cells give rise to many derivatives, including the peripheral nervous system, melanocytes, and a subpopulation of venous endothelial cells (by E13.5) among other cell types (Engleka et al., 2005; Stoller et al., 2008). To exclude a possible contamination of satellite cells with endothelial cells, we performed *Pax3*-lineage tracing using *Pax3*^*Cre*∕+^*; R26*^*mTmG*^ mice (Figure S2B). While adult myogenic cells were mGFP+ (Pax3-Cre recombined), all endothelial cells remained mTOMATO+ (not recombined) (Figure S2B and Movie S1). Moreover, all CD31 (PECAM-1) + endothelial cells were included within the mTOMATO+ population (Figure S2B and Movie S2). These results demonstrate that the *Pax3* lineage does not contribute to skeletal muscle endothelial population, and that skeletal muscle expression of PAX3 is specific to muscle stem cells.

Since *Pax3* is expressed in a subset of the *Pax7*-expressing satellite cells, we compared our gene expression data with previously published datasets of adult muscle stem cells where markers different from PAX3 were used to isolate satellite cells (Figure S3A; Liu et al., 2013; Sinha et al., 2014). *Pax3*-expressing satellite cells were not significantly divergent from previously reported datasets, while embryonic and fetal/early postnatal datasets showed different specific profiles (Figure S3A). Moreover, we compared available data from adult (3–8 month-old) and old satellite cells (18–24 month-old) with our data. We identified a similar variation in all datasets, demonstrating that *Pax3*-expressing satellite cells do not define a subpopulation of satellite cells. Our data therefore are likely representative of the whole satellite population.

Expression profiles from 11 developmental stages were normalized, generating a GEO (GSE63860) showing the kinetics of each transcript over time (Figures 1A,B). Transcript variations were divided into seven clusters based on general expression profiles (Figure 1B and Figure S3B), which were determined to be functionally homogeneous and easily aggregated in defined GO pathways (Figure S3B, Pathways). Furthermore, this *in silico* analysis of the transcriptome through categorization of expression trends (Figure 1B and Figure S3B, Pathways) and specific molecular signatures (Figures 1B–D), yielded known myogenic and related factors (Figure S3B, Genes) (Kuang et al., 2008; Abou-Khalil et al., 2009; Boldrin et al., 2012; Conboy and Rando, 2012). Strikingly, two transition events were revealed: (I) from embryonic to fetal myogenesis (Messina and Cossu, 2009), hypothesized to mark the early onset of satellite cell formation (Kassar-Duchossoy et al., 2005); and (II) the acquisition of quiescence in satellite cells around 3 weeks of age (Figures 1B,C; Lepper et al., 2009; White et al., 2010). These transitions define the three major developmental states: embryonic progenitors (E11.5–E14.5), fetal-to-early postnatal (E15.5–P12) and adult quiescent satellite cells (1–18 months), each with a specific molecular signature (Figures 1B–D). Pairwise comparison between different signatures of up-regulated (UR) and down-regulated (DR) transcripts revealed the genes and pathways defining each developmental period, provided in Figure S4 and Tables S1–S3 (UR), and Tables S4–S6 (DR), respectively. Importantly, this *in silico* analysis also provides new markers for muscle progenitor/stem cell maturation in both UR (extracellular matrix formation, anatomical structure development, immune and inflammatory responses) and DR (cell cycle and DNA repair transcripts or developmental processes) pathways.

The dynamics of our transcriptional profiling reveal that each stage of development is molecularly defined in a more progressive manner than previously recognized.

### Type A-Ephrins and Eph receptors expression during myogenesis

**Figure 2:**
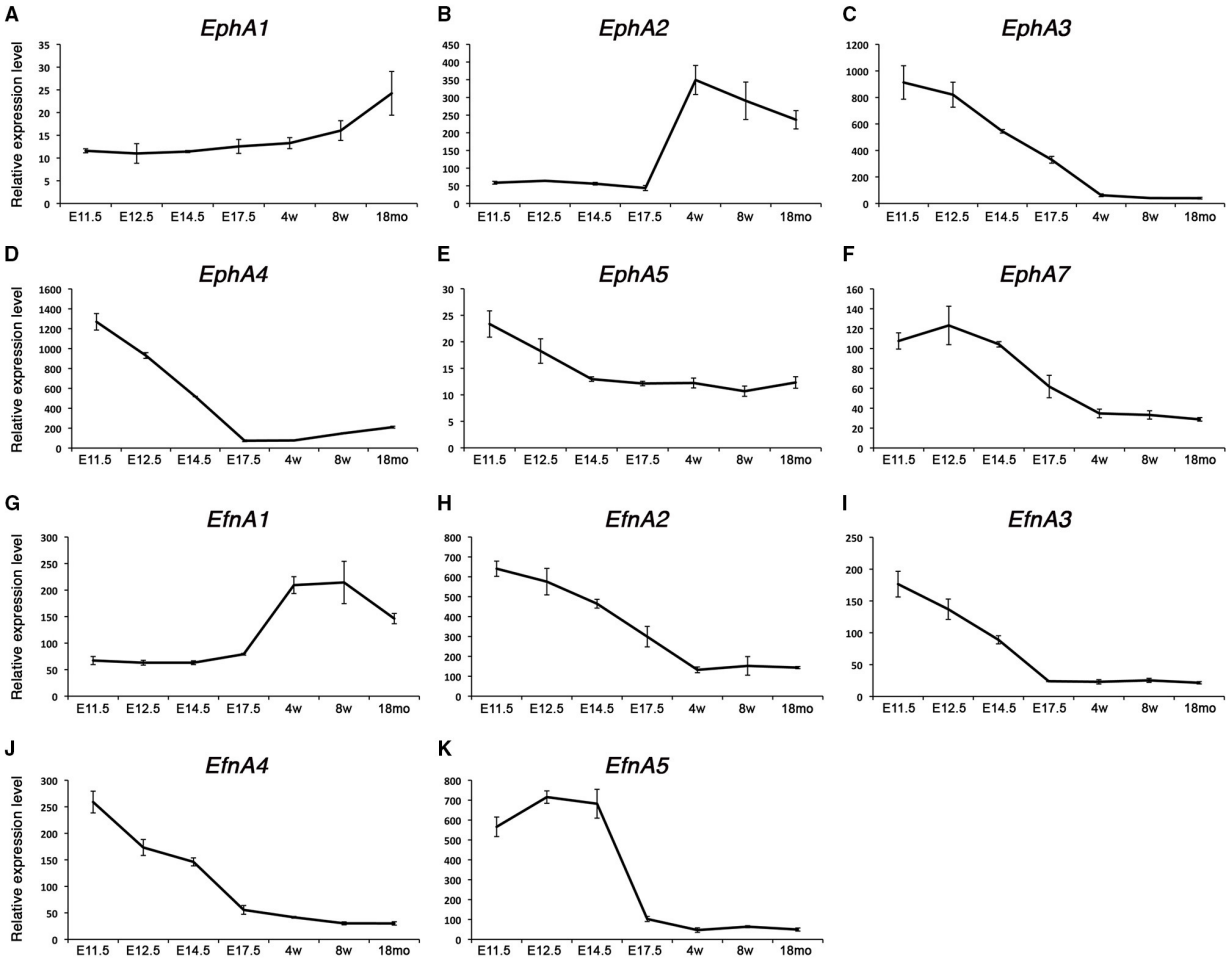
**Expression profile of *EphA* receptors and ephrins in skeletal muscle**. Total RNA from FACS-sorted Pax3-GFP+ cells was used to perform microarray experiments. **(A–F)** Gene expression profiles of type-A Eph receptors from the microarray data during embryonic and postnatal myogenesis. *EphA1* and *EphA2* were up-regulated during the perinatal transition, unlike the rest of the receptors which became down-regulated. **(G–K)** Gene expression dynamics of type-A ephrins during embryonic and postnatal myogenesis. *EfnA1* was up-regulated during the perinatal transition, unlike the rest of the ligands which became down-regulated. *EphA2* and *EfnA1* decline with age. E, Embryonic days; w, age in weeks; mo, age in months.

We have identified a set of transcripts specifically associated with the embryonic and fetal stages of development or the satellite cell lineage. Interestingly, Ephrin family members showed a very dynamic behavior throughout development and postnatal myogenesis, including *EphA*s and *EfnA*s (Figure 2). We could distinguish two distinct behaviors: first, a set of *EphA* transcripts that are up-regulated during the acquisition of muscle stem cell properties (*EphA1* and *EphA2*, Figures 2A,B); second, an independent set that is down-regulated over the same period (*EphA3, EphA4, EphA5*, and *EphA7*, Figures 2C–F). EPHA4 has been reported to bind both EFNA and EFNB ligands subtypes (Singla et al., 2010). This receptor was expressed in the developing embryo, and repressed during postnatal growth (Figure 2D). We found that *EphA4* is strongly expressed during early embryonic development (E11.5) and ceases its expression at the late fetal stage. In our transcriptome data, *EfnA2, EfnA3, EfnA4*, and *EfnA5* ligands expression were also down-regulated during fetal development, being no longer expressed during aging (Figures 2H–K). Interestingly, only *EfnA1* became up-regulated during the perinatal transition that characterizes the emergence of satellite cells (Figure 2G).

### Type B-Ephrins and Eph receptors expression during myogenesis

**Figure 3:**
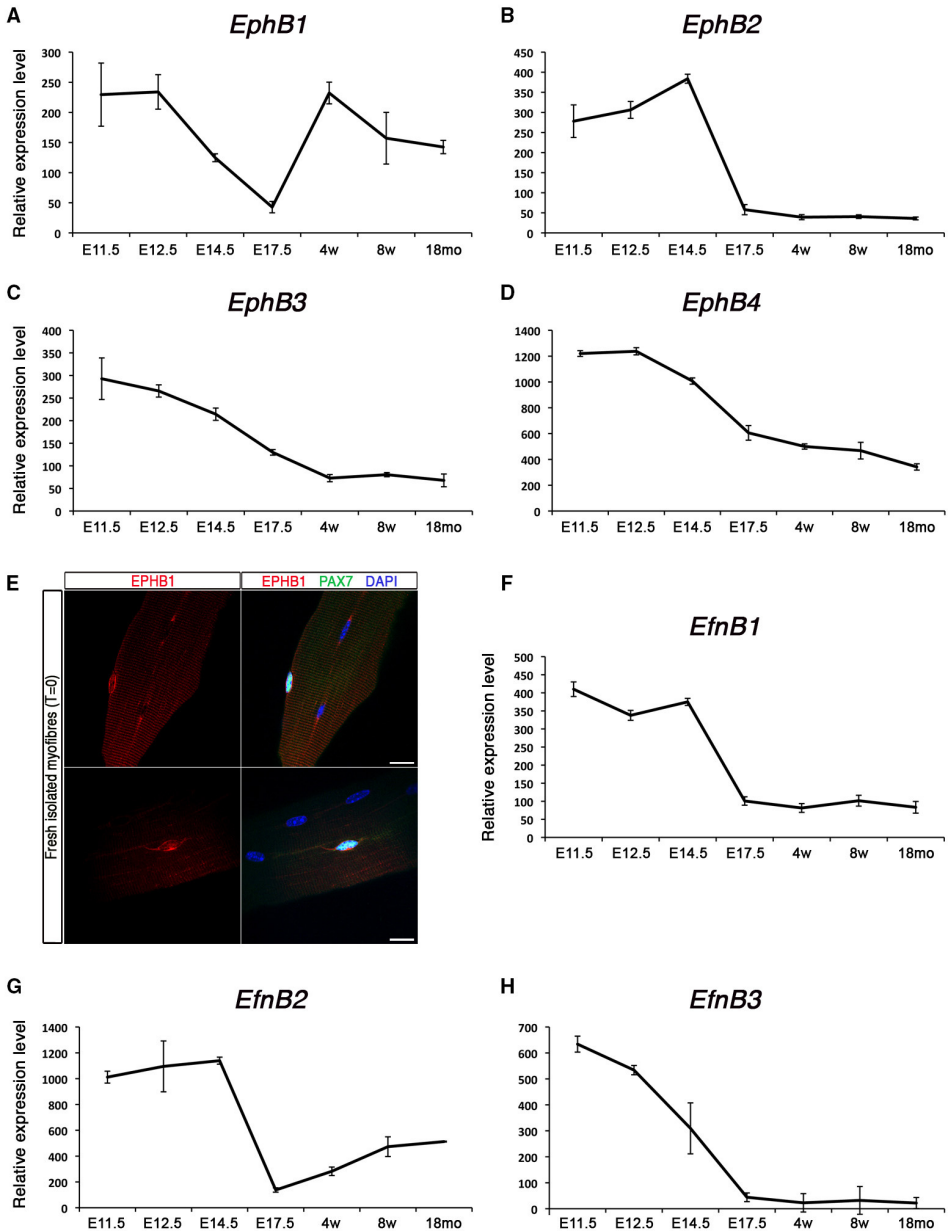
**Expression profile of *EphB* receptors and ephrins in skeletal muscle**. Total RNA from FACS-sorted Pax3-GFP+ cells was used to perform microarray experiments. **(A–D)** Gene expression profiles of type-B Eph receptors during embryonic and postnatal myogenesis. *EphB1* was up-regulated during the perinatal transition, unlike the rest of the receptors which became down-regulated. **(E)** Expression of EPHB1 receptor in quiescent satellite cells on fresh isolated EDL myofibers (T = 0) by co-immunostaining for EPHB1 (red) and PAX7 (green). Nuclei were labeled in blue with DAPI. Scale bars, 10 μm. **(F–H)** Gene expression dynamics of type-B ephrins during embryonic and postnatal myogenesis. All these ephrins were down-regulated during the perinatal transition. E, Embryonic days; w, age in weeks; mo, age in months.

Expression of *EphB*s and *EfnB*s at different stages is shown in Figure 3. Among those, the transmembrane receptor *EphB1* presents a unique dynamic expression profile: initially expressed early during myogenic development, then down-regulated during the fetal stage, and finally re-expressed in postnatal satellite cells (Figure 3A). By contrast, *EphB2, EphB3*, and *EphB4* are highly expressed during early development and progressively repressed as development proceeds (Figures 3B–D). We confirmed that *EphB1* was first expressed during the early stages of embryonic muscle development (Figure S5A), and down-regulated in the fetal stages. While it was weakly expressed in the early immature satellite cells (i.e., P2–P4), it was strongly up-regulated by P14, with expression then maintained, albeit at a lower level, in adult satellite cells. Interestingly, aged satellite cells (18 months old) show a marked decrease in *EphB1* expression (Figure S1A), corresponding to the timing when satellite cells start losing their regenerative capacity (Sousa-Victor et al., 2014).

We used immunostaining on cultured floating myofibers to characterize expression of EPHB1 in muscle stem cells. This culture system recapitulates satellite cell activation, self-renewal and differentiation, similar to the situation observed during muscle regeneration in the adult (Zammit et al., 2004). After 72 h, satellite cells were activated and proliferating (PAX7+ and MYOD+); some cells activated myogenin (MYOG+) and down-regulated PAX7, thus differentiating, and other cells will adopt a divergent fate, withdrawing from cell cycle and maintaining the expression of PAX7 while down-regulating MYOD (Zammit et al., 2004). Co-immunostaining of EPHB1 with PAX7, a specific marker of satellite cells, was observed on isolated fibers (Figure 3E), in 80% of the cells. However, expression was also observed in PAX7, MYOD, and MYOG positive myogenic cells at T = 72 (Figure S5B), demonstrating that EPHB1 was not restricted to quiescent satellite cells, but maintained during the different steps of satellite cell activation and differentiation.

Finally, the kinetics of the ligands for type B-ephrins behaved similarly to most of the type A, being down-regulated during the perinatal transition to the emergence of satellite cells (Figures 3F–H).

### EPHB1 regulates myogenesis in C2C12 cells

C2C12 myoblasts are a classic model to analyze skeletal muscle differentiation (McMahon et al., 1994). Proliferating C2C12 cells were maintained in mitogen-rich medium, but differentiation was induced by switching into a serum poor-medium, thereby inducing MYOG expression and fusion into myotubes. Under long-term differentiation conditions, a reserve cell population emerges that shares some molecular and cellular features with quiescent satellite cells: for example, reserve cells express PAX7, are mitotically quiescent and aligned to the myotubes without fusing (Yoshida et al., 1998; Olguin and Olwin, 2004; Shefer et al., 2006).

**Figure 4:**
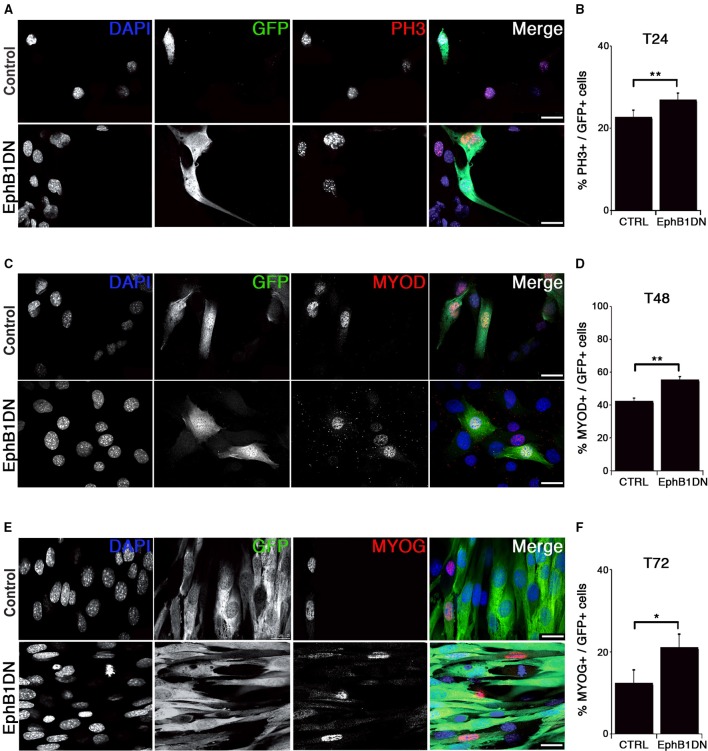
**Impairment of EPHB1 function increases proliferation and myogenic differentiation**. **(A–F)** Overexpression of dominant negative EPHB1 (EphB1DN) using retroviruses in C2C12 cells during proliferation at T = 24 h **(A,B)**, T = 48 h **(C,D)**, and during differentiation at T = 72 h **(E,F)**. For each time point, immunostaining and the corresponding quantifications are shown. Control corresponds to infection by a GFP-expressing empty vector. Nuclei are labeled with DAPI (blue). Cell percentages represent the proportion of transduced cells (GFP+) that co-express PH3 **(B)**, MYOD **(D)**, or MYOG **(F)**. *p*-value ^*^p < 0.05 and ^**^p < 0.01. Minimum number of infected cells was 250, for each marker analyzed. Scale bars, 25 μm.

EPHB1 is expressed in both quiescent and activated satellite cells (Figures 3A,E and Figure S5). The extracellular region of the Eph receptor contains a globular ligand-binding domain, a cysteine-rich region (EGF-like motif), and two fibronectin-type III repeats (Figure S1). The intracellular region contains a tyrosine kinase domain, a SAM (Sterile Alpha Motif) protein-protein interaction domain and a C-terminal PDZ-binding motif (Figure S1A). To assess EPHB1 function in myogenic cells, we generated a dominant negative form of this receptor (EphB1DN) by removing the intracellular domain of the protein (Figure S1B) (Vindis et al., 2003, 2004; Haldimann et al., 2009; Oda-Ishii et al., 2010). Binding of ephrins to Eph receptors induces heterotetramers to initiate the signal cascade, which then will oligomerize and assemble in large signaling clusters (Pitulescu and Adams, 2010). EphB1 truncated receptor (EphB1DN) is therefore able to bind ephrin ligands, but cannot forward signal (Haldimann et al., 2009; Oda-Ishii et al., 2010). We induced expression of EphB1DN or control constructs using retroviral-mediated delivery in the C2C12 myoblastic cell line (Figure 4). EphB1DN was cloned into a modified retroviral vector carrying either an IRES-GFP or CFP to identify transduced cells and packaged using standard methods (Pear et al., 1998; Zammit et al., 2006b). These retroviral constructs were tested in C2C12 and transduction of more than 90% of the cells was observed (Figure S6). Co-staining with EPHB1 antibody showed the expression of the receptor in C2C12 cells (Figure S6A). As our antibody is directed against the last 10 residues of the intracellular domain, a C-terminal 3HA-tagged version of EphB1DN was generated. Figure S6B shows a similar localization to the one of EPHB1 in transduced cells.

We then assayed whether expression of EphB1DN would impact on proliferation of C2C12 cells using an antibody detecting the phosphorylated form of histone H3 at serine 10 (PH3) (Figures 4A,B), and validated by KI67 and EdU incorporation (Figures S7A,B). By 24 h after infection with the EphB1DN-encoding retrovirus, C2C12 cells exhibited a significant increase in the mitotic index, suggesting either a decreased cell cycle time or a decreased myogenic commitment toward differentiation. To further characterize the role of EPHB1 during myogenic differentiation, we analyzed expression of MYOD (Figures 4C,D) and MYOG (Figures 4E,F) in C2C12 cells 48 and 72 h respectively after infection, and found an increased number of cells expressing these myogenic markers. We concluded that EphB1DN leads to increased proliferation and differentiation of C2C12 cells, suggesting a regulatory role for EPHB1 in satellite cell quiescence.

### EPHB1 is required for satellite cell function and renewal

**Figure 5:**
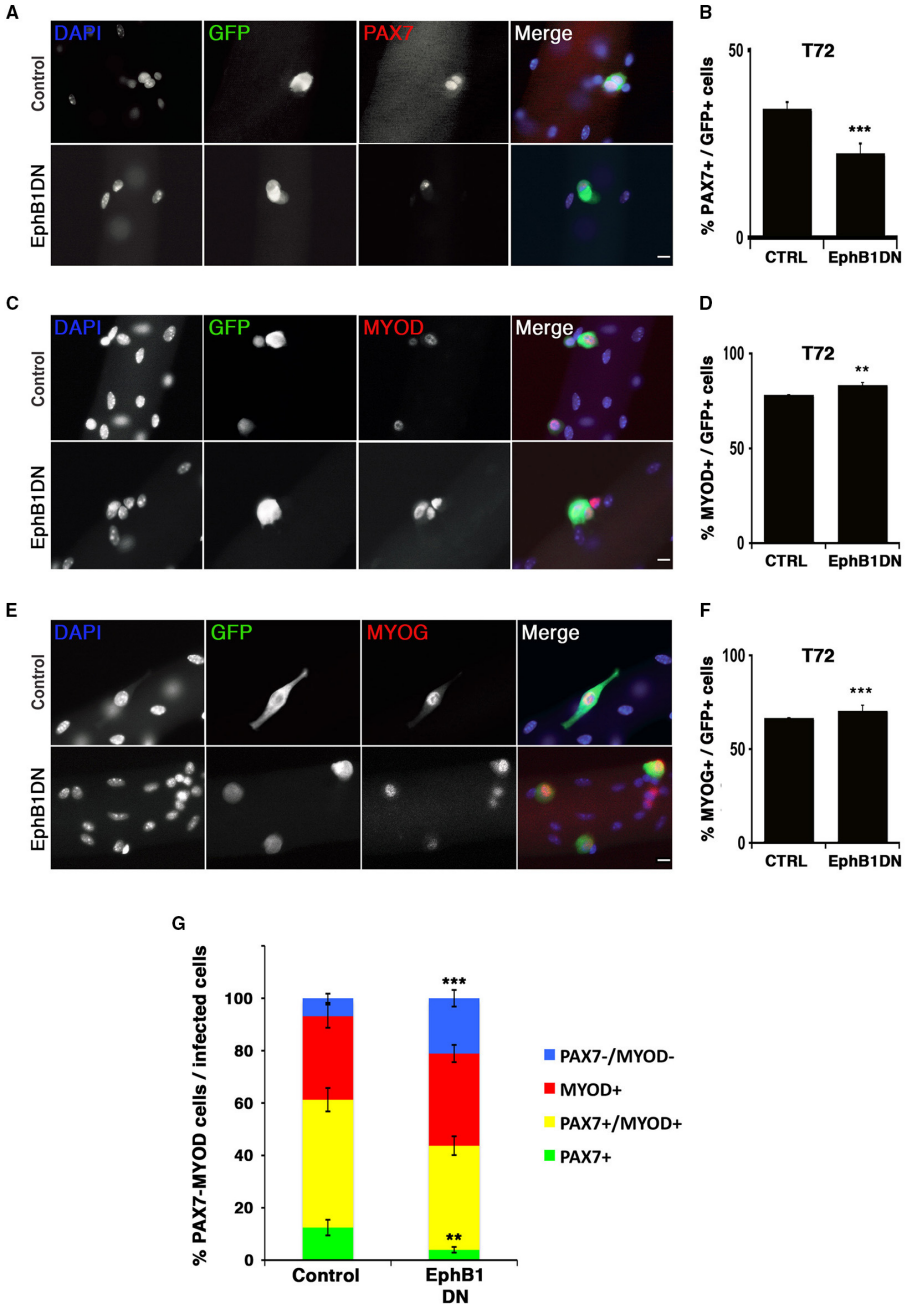
**Impairment of EPHB1 function induces satellite cell differentiation**. Satellite cells were transduced to overexpress a truncated EPHB1 (EphB1DN) after 24 h in culture and analyzed 48 h later (T72). Representative immunofluorescent images are displayed for GFP, DAPI, and PAX7 **(A)**, MYOD **(C)**, and MYOG (Myogenin) **(E)**. Scale bars, 20 μm. Quantifications are illustrated for quiescence/self-renewal **(B)**, activation **(D)**, and differentiation **(F)**. **(G)** Quantification of PAX7 and MYOD satellite cells 48 h after infection with EphB1DN. Control corresponds to infection by a CFP-expressing empty vector. *p*-values: ^**^p < 0.01 and ^***^p < 0.001. For each marker analyzed, the minimum number of infected satellite cells was 200.

We next infected primary satellite cells on muscle fibers in non-adherent cultures to assay the consequence of expressing EphB1DN in activated satellite cells, and assayed self-renewal, proliferation and differentiation (Figure 5). 48 h after infection (72 h post isolation), the number of PAX7+ cells was reduced (Figures 5A,B). Consistently, we observed an increase in the MYOD+ (activated/proliferating and differentiating) population (Figures 5C,D). The number of MYOG+ (differentiating) cells was also increased (Figures 5E,F). Together, these results suggest that EPHB1 is involved in the maintenance of the pool of these adult stem cells, both by promoting self-renewal and reducing activation and differentiation. To appropriately assess self-renewal of satellite cells, Pax7/MyoD co-immunostaining was performed, taking advantage of a retrovirus with a CFP reporter expression restricted to the endoplasmic reticulum and Golgi (Figure 5G and Figures S6, S7). We confirmed that the decrease in the self-renewing satellite cell population (Pax7) correlated to an increase in differentiation (Figure 5G).

### Expression of zinc finger containing proteins during myogenesis

**Figure 6:**
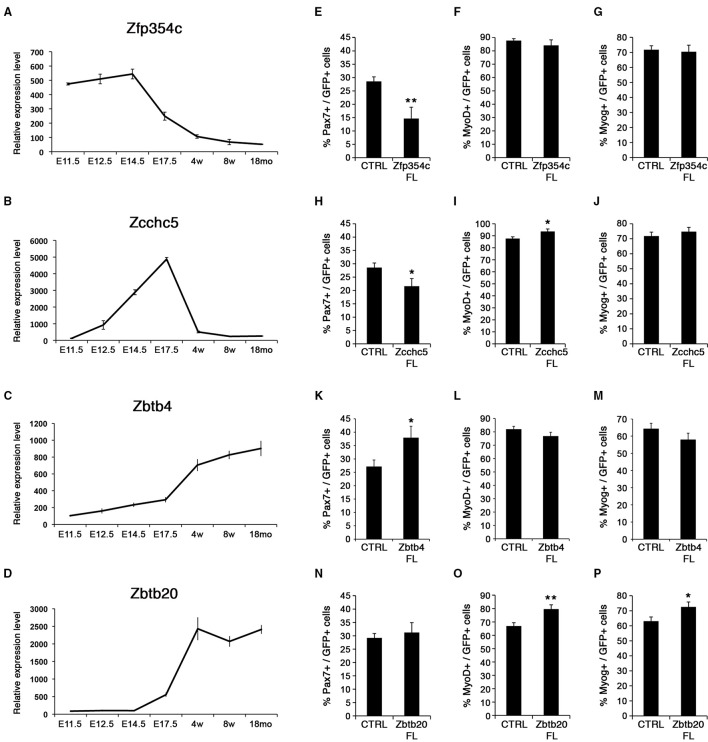
**Zinc finger proteins control satellite cell behavior**. **(A–D)** Expression profiles for zinc finger proteins, *Zfp354c, Zcchc5, Zbtb4*, and *Zbtb20*. Whereas *Zfp354c* and *Zcchc5* are repressed in satellite cells **(A,B)**, *Zbtb4*, and *Zbtb20* are induced during satellite cell formation **(C,D)**. E, Embryonic days; w, age in weeks; mo, age in months. **(E–P)** Quantifications for quiescence/self-renewal (PAX7), activation (MYOD), and differentiation (MYOG) are shown during overexpression of the different zinc fingers: *Zfp354c* **(E–G)**, *Zcchc5* **(H–J)**, *Zbtb4* **(K–M)**, and *Zbtb20* **(N–P)**. All analyses were performed 48 h after infection. *p*-value ^*^p < 0.05 and ^**^p < 0.01. For each marker analyzed, the minimum number of infected satellite cells was 200.

Candidate genes coding for ZFP354c and ZCCHC5 zinc finger containing proteins were repressed during the emergence of satellite cells around birth (Figures 6A,B). Down-regulation of these factors was observed in muscle progenitors at the fetal stage overlapping with the emergence of satellite cells. These two zinc finger containing-proteins were not expressed in adult and aged satellite cells. While *Zfp354c* was highly expressed during early myogenesis and gradually repressed from fetal stages (Figure 6A), *Zcchc5* was not expressed during early embryonic myogenesis (Figure 6B), but appeared during early establishment/formation of the satellite cell pool, before being completely down-regulated during acquisition of satellite cell quiescence. According to the known functions of these factors, we can hypothesize their possible involvement during MPCs proliferation (*Zfp354c*), or for a correct determination of the MPC fate to become the muscle stem cells (*Zcchc5*).

By contrast, two other zinc finger containing proteins *Zbtb4* and *Zbtb20*, were not expressed during development but were induced during establishment of satellite cells and acquisition of quiescence (Figures 6C,D). Moreover, high expression of these zinc finger containing-proteins was maintained in adult and aged satellite cells, implicating a possible function in maintaining quiescence of muscle stem cells. Strikingly, these factors are induced during cardiotoxin-induced muscle regeneration *in vivo* (Figure S8A).

### Effect of zinc finger containing proteins in postnatal satellite cells

We manipulated expression of *Zfp354c, Zcchc5, Zbtb4, and Zbtb20* using retroviral-mediated delivery in isolated myofiber cultures as above. We generated vectors carrying either a full-length transcript for overexpression, or dominant negative forms to analyze function.

Overexpression in satellite cells of either *Zfp354c* or *Zcchc5* maintained expression in satellite cells that no longer expressed the endogenous gene (Figures 6E–J). Notably, overexpression of *Zfp354c* led to a decreased number of PAX7+ satellite cells compared to control (Figure 6E) with no apparent effect during activation (MYOD+) and differentiation (MYOG+) (Figures 6F,G). These results demonstrate that overexpression of *Zfp354c* resulted in a reduction of self-renewal capacity of the satellite cells.

Overexpression of *Zcchc5* in satellite cells, as with *Zfp354c*, resulted in a decrease of the PAX7+ population relative to control (Figure 6H). Strikingly, the proportion of MYOD+ satellite cells increased without affecting MYOG-expressing differentiated cells (Figures 6I,J). These results showed that overexpression of *Zcchc5* induced decreased self-renewal promoting the proliferation of satellite cells. Our functional data is consistent with a specific requirement of *Zcchc5* function during the growth phase where production of MPC is needed.

We next overexpressed the other two BTB-containing zinc finger factors, *Zbtb4* and *Zbtb20* (Figures 6K–P). *Zbtb4* increased PAX7+ satellite cells (Figure 6K), whereas *Zbtb20* promoted myogenic progression by increasing the activated/proliferating (MYOD+; Figure 6O) and differentiating (MYOG+; Figure 6P) populations. These results suggested that these transcriptional repressors might be required for specification/maintenance of the muscle stem cell pool. Strikingly, inhibiting function by expression of ZBTB4 dominant negative constructs, missing the POZ DNA-binding domain, displayed an increase in satellite cell differentiation (MYOG+) without affecting the activated/proliferating population (MYOD+) (Figures S8B,C). On the other hand, ZBTB20 could behave with a previously described phenotype in the brain of *Zbtb20* transgenic mice (Nielsen et al., 2007), where overexpression of ZBTB20 represses cell fate transitions in newborn pyramidal neurons. Moreover, overexpression of ZBTB20 has been recently described as a prognostic marker by promoting tumor growth of human hepatocellular carcinoma (Kan et al., 2016). Thus, ZBTB20 could be regulating muscle regeneration during satellite cell activation as suggested in Figure S8A.

### *Hmga2* must be repressed for appropriate satellite cell function

**Figure 7:**
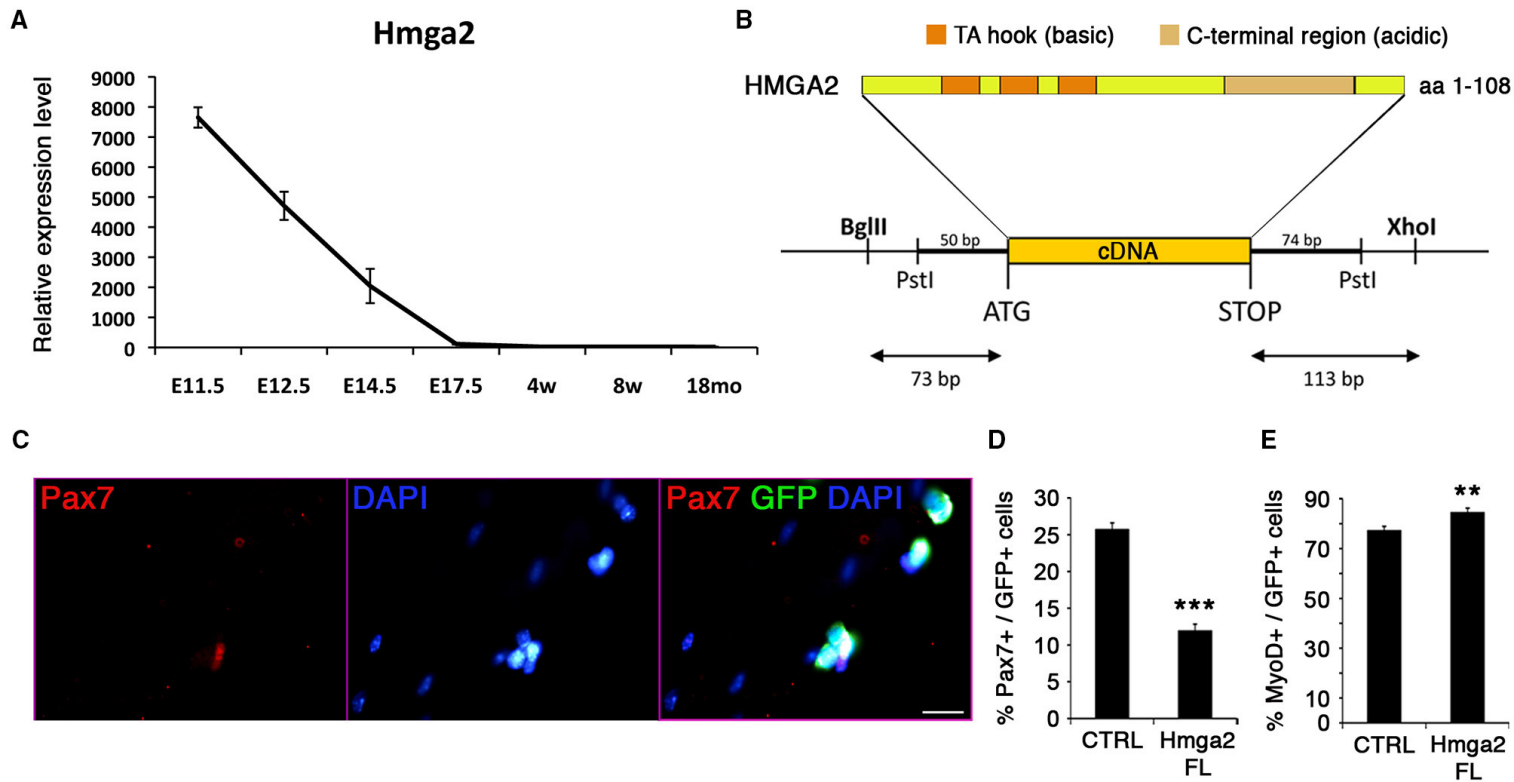
**HMGA2 reduces the pool of satellite cells**. **(A)** Expression profile of *Hmga2* during development. E, Embryonic days; w, age in weeks; mo, age in months. **(B)** Protein structure of the non-histone, DNA-binding chromatin HMGA2 factor containing three DNA-binding sites (AT hook motifs) and the basic terminal region, which can bind various proteins. **(C)** Representative image for the co-immunofluorescence of GFP (green) and PAX7 (red) with DAPI counterstain (blue). Quantification corresponds to the analysis of quiescence **(D)** and activation **(E)** of the satellite cells. Forty eight hours after infection, retroviral-mediated overexpression of HMGA2 caused a reduction in the population of PAX7+ cells **(D)** and an increase MYOD+ cell population **(E)**. *p*-value ^**^p < 0.01 and ^***^p < 0.001. For each marker analyzed, the minimum number of infected satellite cells was 500. Scale bar, 20 μm.

*Hmga2* was highly expressed during early development (Figure 7A), when MPCs expand to populate the future skeletal muscle of the body. As development proceeds, *Hmga2* was no longer expressed, and was not detected in the emerging satellite cells prior to birth. Nishino and collaborators have described a similar behavior where *Hmga2* is highly expressed in fetal neural stem cells and declining with age (Nishino et al., 2008).

We analyzed the effect of overexpressing *Hmga2* in satellite cells (Figures 7B–E). A retroviral construct carrying full-length cDNA including the coding sequence for the basic and acidic region of the protein was generated (Figure 7B), and satellite cells transduced using isolated myofiber culture. *Hmga2* overexpression led to a strong reduction in the pool of satellite cells expressing PAX7 (Figures 7C,D), with an increase on the activated/proliferating MYOD+ muscle stem cells (Figure 7E); data consistent with the work from Li and colleagues describing HMGA2 as a regulator of myoblast proliferation by direct interaction with the RNA-binding protein IGF2BP2 (Li et al., 2012).

## Discussion

PAX3 and PAX7 are key upstream regulators of skeletal myogenesis (Relaix et al., 2005; Buckingham and Relaix, 2015). Postnatally, while PAX7 labels all satellite cells (Seale et al., 2000), PAX3 is maintained in a subset of these adult muscle stem cells (Relaix et al., 2006). A complex balance between extrinsic cues and intrinsic regulatory mechanisms is needed to tightly control satellite cell determination and function. For example, defects in satellite cell regulation or changes in their niche, such as during postnatal growth or in degenerative conditions and aging, can impair muscle regeneration with possible fatal consequences (Dumont et al., 2015). Hence, identifying and manipulating muscle progenitor stem cells, and understanding the mechanisms underlying cell fate decision and self-renewal (Relaix, 2006; Boutet et al., 2007) are essential for development of stem cell-based therapeutic strategies.

We have developed a FACs-based chronological transcriptome profile of myogenic stem cells, sampled from embryonic and fetal progenitors, to postnatal, adult, and aging satellite cells. This provides a comprehensive description of gene expression changes throughout life of muscle stem cells and identifies two important transition events, which delimit three developmental periods of muscle stem cells with specific molecular signatures: (1) embryonic, (2) fetal to early proliferating postnatal progenitors, and (3) quiescent adult muscle stem cells (Buckingham and Relaix, 2007; Braun and Gautel, 2011). The intersection between specifically expressed genes and functional pathways defines a molecular signature unique to each developmental period. As such, our study is instrumental for a better understanding of both myogenesis and the establishment and maintenance of quiescent adult stem cells.

The dynamics of our transcriptional profiling reveal that cellular processes characterizing muscle stem cells, including transition from the fetal lineage to postnatal stem cells, establishment of quiescence and formation of a functional niche, are defined molecularly in a more progressive manner, highlighting that establishment of the satellite cell lineage is more gradual than previously recognized. For example, cell division processes (i.e., cyclins such as *Ccne1/2* or cyclin-dependent kinases such as *Cdk1*) were gradually down-regulated throughout the second transition, corresponding to the entry into satellite cell quiescence and consistent with analysis of fetal progenitor cell proliferation (Picard and Marcelle, 2013). At the same time, known satellite cell markers such as *Sdc4* (Syndecan 4), *Itga7* (Integrin Alpha-7) or *Cav1* (Caveolin 1) were progressively up-regulated (Cornelison, 2001; Gnocchi et al., 2009).

**Figure 8:**
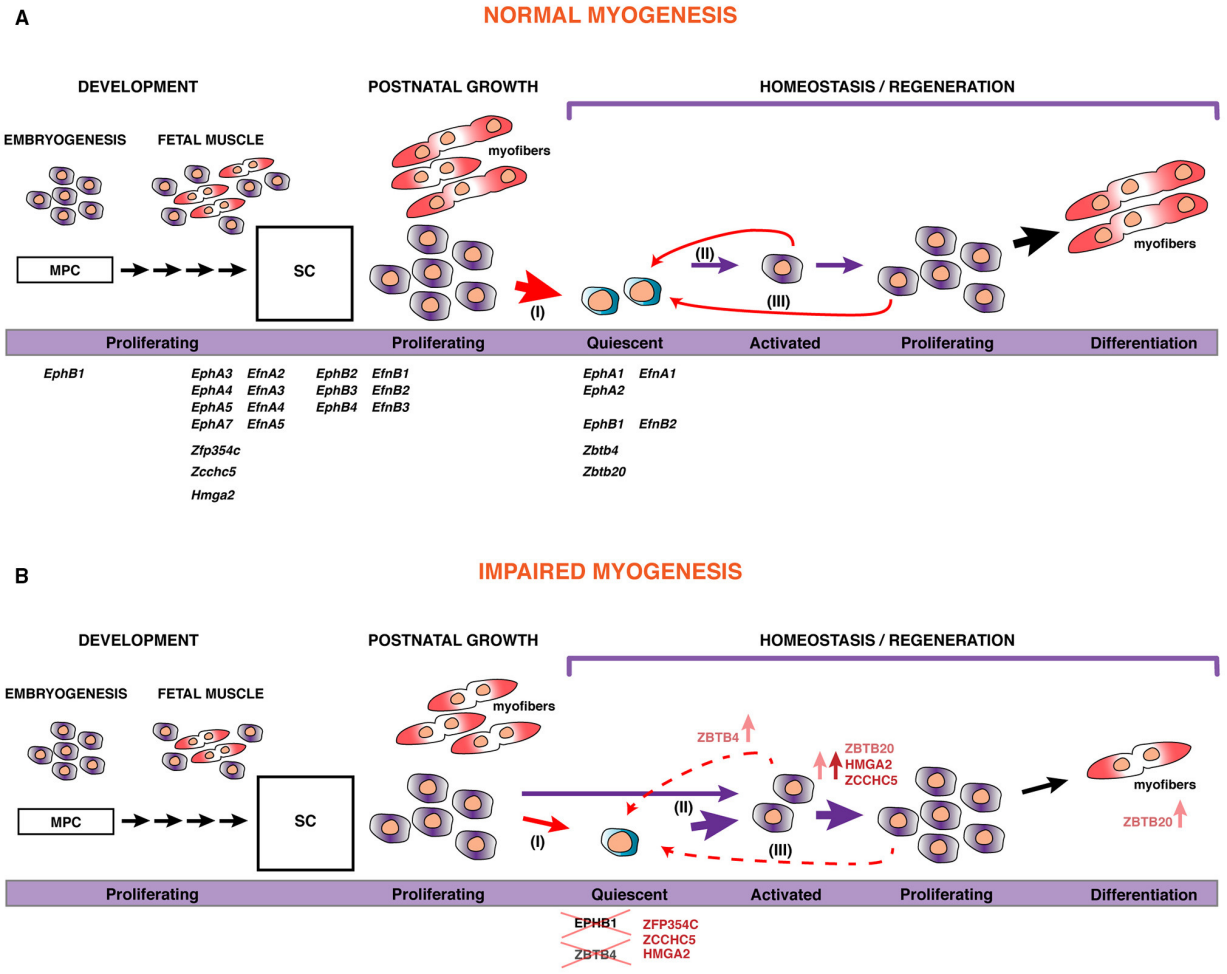
**A model for skeletal muscle stem cell behavior during myogenesis**. **(A)** During development, embryonic and fetal progenitors are highly proliferative, augmenting the pool of MPC that will differentiate and fuse to form myofibers. As development proceeds, some of these MPCs become satellite cells (SC), the postnatal muscle stem cells. During the perinatal transition, many of these SCs will contribute to the maturation of myofibers, with a pool of stem cells maintained within their natural niche, underneath the basal lamina surrounding the myofiber. Satellite cells become quiescent by 3 weeks after birth (I). However, in response to injury or disruption of the basal lamina, SCs become activated (II), start proliferating, and differentiate to fuse with each other or to existing myofibers for repair. Some of these will self-renew to replenish the pool of quiescent stem cells (III). The putative role and expression of the set of transcripts that we identified in this work is displayed. In addition, we show that manipulating their expression or function can lead to impaired myogenesis **(B)**, either via reduction of the pool of stem cells by promoting proliferation or cell fate determination, or through inducing precocious myogenic differentiation.

From this large-scale myogenesis transcriptome, we functionally characterized a set of genes to provide novel intrinsic factors that regulate satellite cell behavior (Figure 8A).

### Eph/Ephrin pathway and myogenesis

EPHB1 is not only involved in motility and guidance in skeletal muscle cells as previously shown (Stark et al., 2011), but also acts as a novel regulator of myogenesis. Our findings point to a function during self-renewal of satellite cells, since a dominant negative form of EPHB1 led to increased proliferation and differentiation in C2C12 myogenic cells and satellite cells in the myofiber experimental model. The increase in cell differentiation is achieved at the expense of self-renewal of the satellite cell population (Figure 8B). Identifying the molecular regulators of satellite cell renewal is important since it was recently shown that targeted depletion of the satellite cell pool leads to complete impairment of muscle regeneration following injury (reviewed in Relaix and Zammit, 2012).

Eph/ephrin signaling takes place via direct cell-cell interaction; either as *trans* or *cis* signaling (Arvanitis and Davy, 2008; Pitulescu and Adams, 2010). This interaction could take place with the muscle fiber, between satellite cells, or via interactions with other cell types in the microenvironment (i.e., macrophages and/or microvascular cells). The satellite cell population is heterogeneous, with specific markers labeling subpopulations of the satellite cell pool and different myogenic behaviors *in vivo* or *ex vivo* (Relaix et al., 2006; Kuang et al., 2007; Rudnicki et al., 2008; Ono et al., 2010; Rocheteau et al., 2012). Whether arising through lineage or stochastic events, more “stem” satellite cells likely correspond to independently identified label-retaining satellite cells during growth and after injury (Shinin et al., 2006; Rocheteau et al., 2012; Chakkalakal et al., 2014), or displaying different rates of cell division (Ono et al., 2012). Interestingly, satellite cells can asymmetrically divide and it will be of interest to evaluate if interaction between fibers and/or the satellite cells via the Eph/ephrin signaling plays a role in these cell fate decisions. Finally, our results are consistent with the work from Chumley and colleagues, showing that proliferative neuronal progenitor cells increase in *EphB1* mutant mice (Chumley et al., 2007), thereby demonstrating an important role of EPHB1 in maintenance of neuronal progenitors in the quiescent state.

Eph/ephrin signaling has also been shown to play a role in regulating other stem cell niches, for instance in the dental (Stokowski et al., 2007) or osteochondral (Arthur et al., 2011) system. Using an ephrin “stripe” assay revealed that satellite cells respond to a subset of ephrins with repulsive behavior *in vitro* (Stark et al., 2011). Our finding that EPHB1 is also regulating myogenesis suggests that this guidance signaling might impact multiple aspects of muscle regeneration, including escape from the niche, directed migration to sites of injury, cell-cell interactions among satellite cell progeny, and differentiation and patterning of regenerated muscle.

### Identification of novel zinc finger proteins regulating myogenesis

We identified a set of zinc finger containing proteins with a dynamic expression profile during myogenesis. We have shown that overexpression of *Zfp354c* decreased self-renewal of satellite cells (summarized in Figure 8). In the skeletal system, the highest *Zfp354c* expression is in proliferating bone cells compared to mature and differentiated chondrocytes. Interestingly, ZFP354C is induced as an early response to BMP-7 (Jheon et al., 2001). It has been shown that overexpression of *Zfp354c* affects osteoblast differentiation, a lineage that is also regulated by BMP signaling (Jheon et al., 2001). Moreover, overexpression of this gene results in a decrease in osteogenic differentiation by suppressing BMP-7 induced alkaline phosphatase activity, an early marker of osteogenesis (Jheon et al., 2003). Furthermore, BMP signaling prevents myogenic differentiation of satellite cells, and is also involved in regulation of satellite cells during proliferation or differentiation (Friedrichs et al., 2011; Ono et al., 2011). In essence, there is strong evidence of a functional interaction between ZFP354C and BMP7, though the precise relationship between the two proteins is not fully understood (Jheon et al., 2002). Future studies will be necessary to evaluate whether a functional interaction between ZFP354C and BMP7 regulates myogenesis and, in general, to identify the downstream gene regulatory networks for all four zinc finger proteins presented here, ZFP354C, ZCCHC5, ZBTB4, and ZBTB20, which are able to strongly repress transcription of target genes. These zinc fingers, thus, could be used as potentially powerful tools for regulation of muscle stem cell function.

### HMGA2 function and its role in myogenesis and satellite cell fate decision

HMGA2 is a co-regulator of chromatin structure and pluripotency in stem cells (Pfannkuche et al., 2009). The role of HMGA2 in myoblast proliferation has been previously described in neonatal and regenerating muscle (Li et al., 2012). *Hmga2* is sharply induced during satellite cell activation. We found that *Hmga2* is highly expressed during early muscle development and progressively down-regulated in the fetal stages, while it is not expressed during growth or aging (Figure 7A). It has been shown that *Hmga2* knockout mice are smaller and show defects in postnatal skeletal muscle (Zhou et al., 1995; Li et al., 2012). In addition, HMGA2/IGF2BP2 has been shown to be critical for myoblast proliferation and early myogenesis, but should be down-regulated in order for myoblasts to differentiate into multinucleated skeletal muscle. Indeed, when satellite cells are activated and entering cell cycle, HMGA2 is up-regulated and activates the expression of IGF2BP2 (Li et al., 2012). Our transcriptome analysis shows that *Igf2bp2* behaves similarly to *Hmga2* before birth, but in contrast to *Hmga2, Igfbp2* is induced in adult stem cells, including aged satellite cells (data not shown). This suggests that IGF2BP2 could be functionally independent of HMGA2 in adult and aged satellite cells.

In conclusion, understanding the molecular signals that control and regulate the muscle stem cell population is essential to identify new therapeutic strategies for muscle diseases. Here we provide a set of potential new regulators of myogenesis that improves the understanding and knowledge of the intrinsic factors controlling muscle stem cell acquisition, establishment, maintenance and function in the adult, and could be targeted to modify the regenerative capacity of endogenous skeletal muscle stem cells.

## Author contributions

SAM, AR, and JM designed and performed experiments, and analyzed data. SAM wrote the manuscript. AD analyzed bioinformatic data. DM, FA, and TC performed experiments. PZ designed experiments. FR oversaw the entire project, designed experiments, analyzed data and wrote the manuscript. All authors read and approved the final manuscript.

### Conflict of interest statement

The authors declare that the research was conducted in the absence of any commercial or financial relationships that could be construed as a potential conflict of interest.
