# Supplementary material for: Gene Expression Profiling of Muscle Stem Cells Identifies Novel Regulators of Postnatal Myogenesis
Source: Front Cell Dev Biol. 2016 Jun 21;4:58. doi: 10.3389/fcell.2016.00058 (PMC4914952; doi:10.3389/fcell.2016.00058)
Supplement: Supplementary file 9 [file DataSheet1.PDF]

Supplementary Movie Legends

Movie S1: 3D-reconstruction of mGFP and mTOMATO localization in skeletal muscle of *$Pax3^{Cre/+}$;$R26^{mT\text{-}mG}$* mice. Movie of a collection of 0.3 nm captures in Z-stack of a *Soleus* muscle cryosection. mGFP is exclusively expressed in muscle and mTOMATO in the non-myogenic contributors of skeletal muscle. 40x magnification.

Movie S2: **3D-reconstruction of mTOMATO and PECAM-1 (CD31) colocalization in skeletal muscle of *$Pax3^{Cre/+}$;$R26^{mT\text{-}mG}$* mice.** Movie of a collection of 0.3 nm captures in Z-stack of a *Soleus* muscle cryosection. mTOMATO non-myogenic cells include all endothelial cells (PECAM-1+) within skeletal muscle. 40x magnification.

Supplementary Table Legends

Supplementary Table 1: UP-REGULATED GENES (FC>2)

Affymetrix transcriptome analysis specific for Pax3+ embryonic cells.

Supplementary Table 2: UP-REGULATED GENES (FC>2)

Affymetrix transcriptome analysis specific for Pax3+ fetal/early postnatal cells.

Supplementary Table 3: UP-REGULATED GENES (FC>2)

Affymetrix transcriptome analysis specific for Pax3+ adult quiescent satellite cells.

Supplementary Table 4: DOWN-REGULATED GENES (FC<1/2)

Affymetrix transcriptome analysis specific for Pax3+ embryonic cells.

Supplementary Table 5: DOWN-REGULATED GENES (FC<1/2)

Affymetrix transcriptome analysis specific for Pax3+ fetal/early postnatal cells.

Supplementary Table 6: DOWN-REGULATED GENES (FC<1/2)

Affymetrix transcriptome analysis specific for Pax3+ adult quiescent satellite cells.

All tables contain the list of genes that are specifically up- (UR) or down-regulated (DR) in the different signatures described in the study: embryonic, fetal-early postnatal, and adult stem cells. FC indicate de fold change were the cut off was established to determiner up- or down-regulation.
