# Supplementary material for: Gene Expression Profiling of Muscle Stem Cells Identifies Novel Regulators of Postnatal Myogenesis
Source: Front Cell Dev Biol. 2016 Jun 21;4:58. doi: 10.3389/fcell.2016.00058 (PMC4914952; doi:10.3389/fcell.2016.00058)
Supplement: Supplementary file 10 [file Image1.PDF]

AlonsoMartinS_Supp fig1.

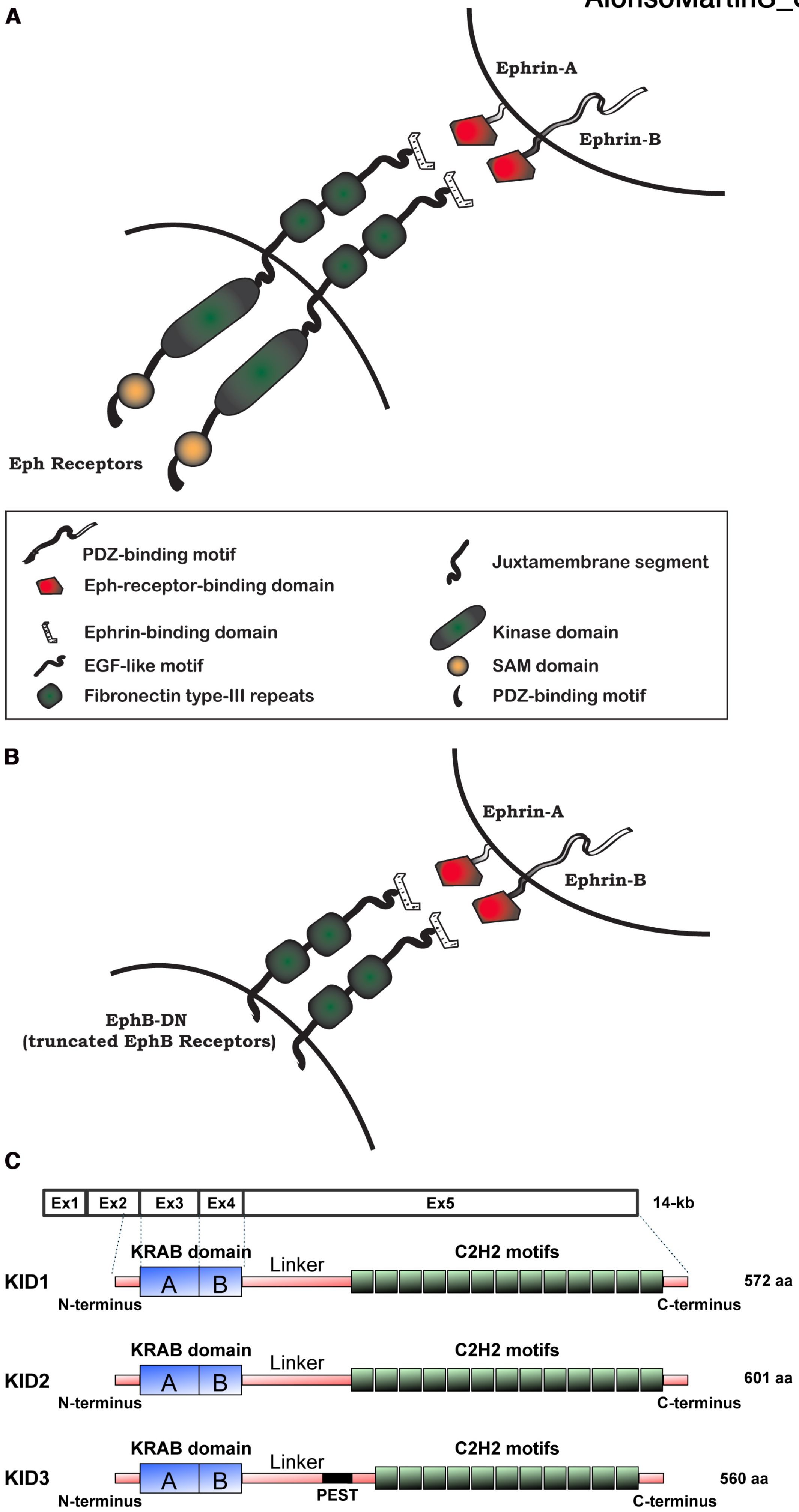

FIGURE S1: Structure of ephrin, Eph receptors and KID family of zinc finger proteins. (A) Ephrin A and B share an extracellular domain containing the binding domain to their receptors. Ephrin-B unlike ephrin-A ligands possess a short cytoplasmic domain. The specificity of the receptors depends on their ephrin binding domains. The extracellular domain consists of these binding domains, an EGF-like motif and two fibronectin type-III repeats. The signal is transduced into the intracellular space via the kinase and SAM domains and the C-terminal PDZ-binding motif. (B) Modified dominant negative version of EphB receptors (EphB-DN). The resulting truncated protein is defective in its C-terminus, missing its cytoplasmic domain responsible of signaling, while preserving the binding to the ligands. (C) The *Kid* gene family encodes C2H2 zinc finger transcription factors with a highly conserved Kruppel-associated box (KRAB) domain at its N-terminus. The KRAB domain can be subdivided into KRAB A and KRAB B boxes. Linker is the unique spacer region linking the KRAB and zinc finger domains. KID3 is also known as ZFP354C or AJ18. In this protein, there is a possible PEST protein instability sequence, suggesting a rapid turnover in vivo (underlined). Ex, exon; aa, amino acids.
