# Supplementary material for: Gene Expression Profiling of Muscle Stem Cells Identifies Novel Regulators of Postnatal Myogenesis
Source: Front Cell Dev Biol. 2016 Jun 21;4:58. doi: 10.3389/fcell.2016.00058 (PMC4914952; doi:10.3389/fcell.2016.00058)
Supplement: Supplementary file 11 [file Image2.PDF]

AlonsoMartinS_Supp fig2.

A

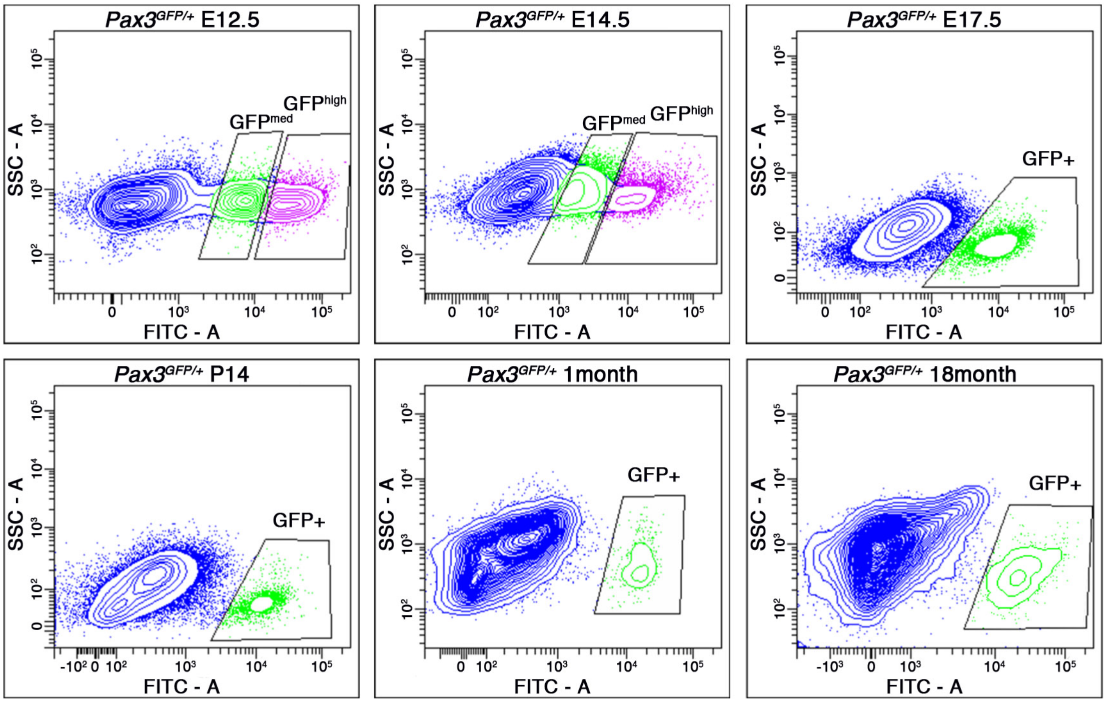

B

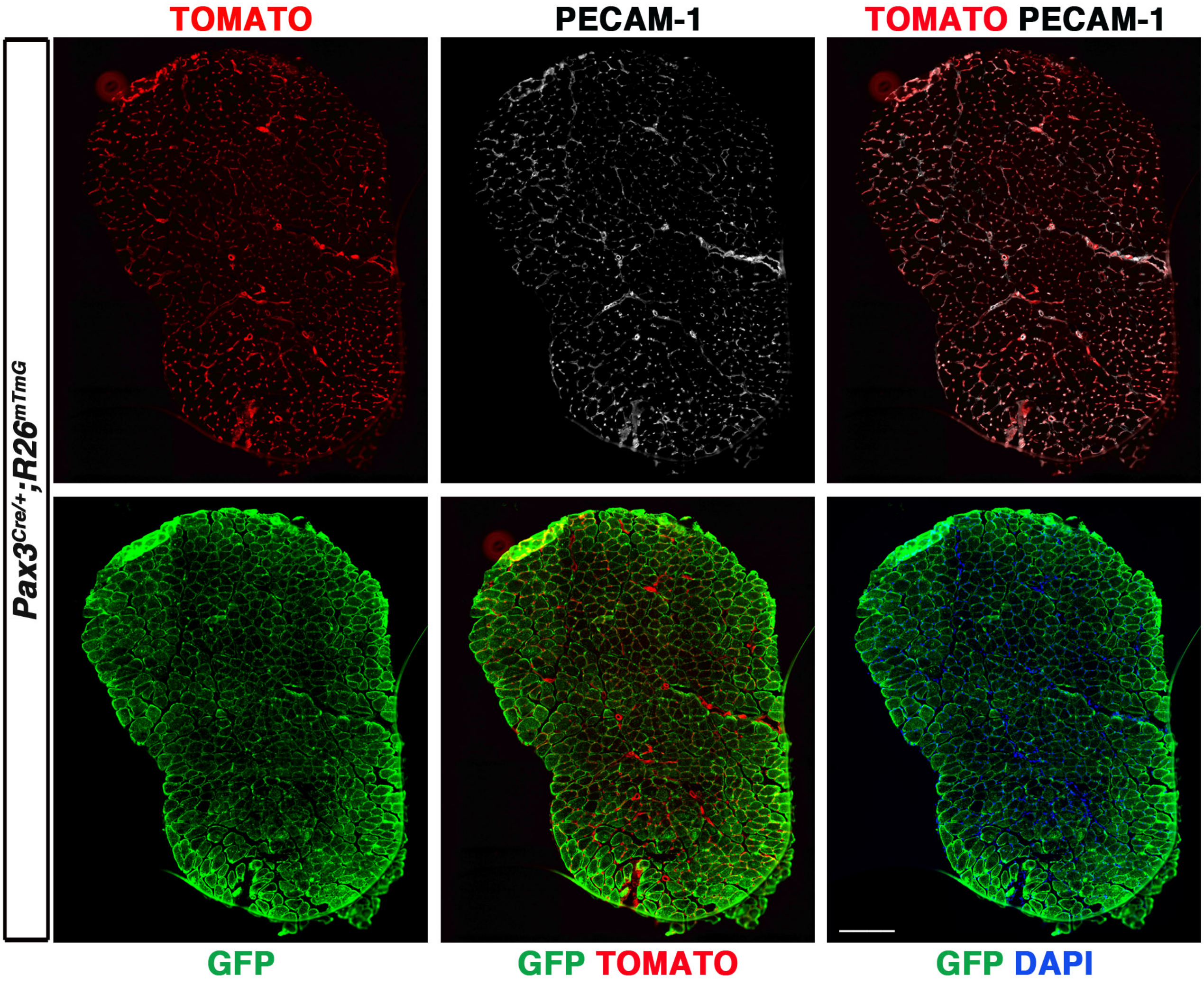

FIGURE S2: Pax3GFP+ cells do not contribute to skeletal muscle vascular endothelium. (A) FACS-sorting profiles from *Pax3*$^{GFP/+}$ hypaxial somites and skeletal muscles for different ages during development and after birth are shown. Embryonic stages up to E15.5 show two separate GFP populations: GFP^{high} containing the melanocyte population and GFP^{med} the muscle progenitor cells. After E15.5 (skin easily removed) only a single GFP population is observed. The percentage of GFP+ cells drops in aged animals, suggesting a progressive decline in the muscle stem cell population. E, Embryonic days; P, Postnatal days. (B) Representative *Soleus* muscle cryosections images of adult *Pax3*$^{Cre/+}$*;R26*$^{mT\text{-}mG}$ mouse. PAX3 lineage tracing was performed crossing *Pax3*$^{Cre/+}$ with *R26*$^{mT\text{-}mG}$ mice. Only Pax3-derived cells that have been Cre-recombined during development are expected to express mGFP, while the other cells will keep expressing mTOMATO. Indeed, GFP staining confirms that skeletal muscle originates from PAX3-derivatives, whereas colocalization of mTOMATO with PECAM-1 (CD31) shows that mTOMATO labels cells of different lineages, most likely endothelial and hematopoietic cells. Scale bar, 200 µm.
