# Supplementary material for: Gene Expression Profiling of Muscle Stem Cells Identifies Novel Regulators of Postnatal Myogenesis
Source: Front Cell Dev Biol. 2016 Jun 21;4:58. doi: 10.3389/fcell.2016.00058 (PMC4914952; doi:10.3389/fcell.2016.00058)
Supplement: Supplementary file 12 [file Image3.pdf]

A

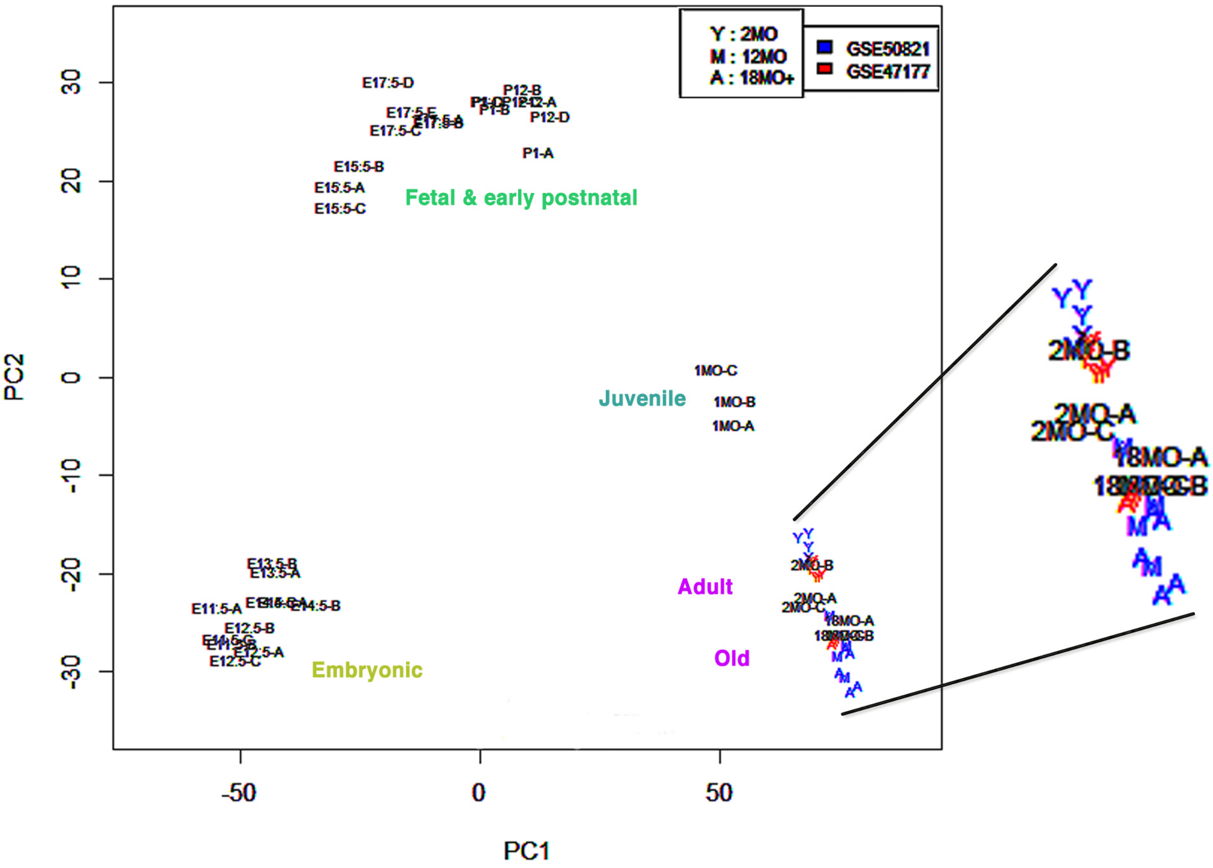

B

Clusters (Total No. of genes)	Myogenic & related genes (represented in cluster)	Representative functional pathways
**a (1029)**	*Angpt1, Fst, Wnt11*<br>*Ephb1, Efnb2*<br>*Smad6, Smad7*	GO:0048856 - anatomical structure development<br>GO:0001944 - vasculature development<br>Novel regulators of myogenesis
**b (872)**	*Myog, Cdkn1c, Cdk1, Cdk4*<br>*Ccna2, Ccnb1, Ccnb2, Ccne1, Ccne2*<br>*Epha3, Efna2, Zfp354c, Zcchc5*	GO:0007049 - cell cycle<br>KEGG_mm04110 - cell cycle<br>Novel regulators of myogenesis
**c (3426)**	*Sox2, Sox4*<br>*Peg3, Ctnnb1, Hoxd1*<br>*Ephb2, Ephb4, Efnb1*	GO:0006396 - RNA processing<br>GO:0006281 - DNA repair<br>Novel regulators of myogenesis
**d (409)**	*Epha4, Efna3, Efna4, Efna5,*<br>*Ephb3, Efnb3, Hmga2, Ccnd2, Foxd1*<br>*Axin2, Wnt2, Wnt5a, Lef1, Fzd2*	GO:0007399 - nervous system development<br>GO:0001501 - skeletal system development<br>Novel regulators of myogenesis
**e (3284)**	*Ccna1, Ccnb3, Ccnd3*<br>*Six1, Six4, Wnt1, Wnt7b*	GO:0007519 - skeletal muscle tissue development<br>GO:0060538 - skeletal muscle organ development
**f (410)**	*Pax3, Pax7, Myf5, Cav1*<br>*Cdkn1a, Notch1, Notch3, Itga7*<br>*Zbtb4, Zbtb20*	GO:0031012 - extracellular matrix<br>GO:0007517 - muscle organ development<br>Novel regulators of myogenesis
**g (3352)**	*Cdkn1b, Sdc4*<br>*Sox7, Sox17, Sox18*<br>*Epha2, Efna1*	GO:0006955 - immune response<br>GO:0006954 - inflammatory response<br>Novel regulators of postnatal myogenesis

FIGURE S3: Validation of PAX3 as a marker for skeletal muscle cell sorting and summary of the microarray data analysis. (A) PCA analysis comparing our microarray data with other published datasets emphasizing the match in adult and old satellite cells (see magnification). Previously used markers were CD45-TER119-SCA1-CD29+CXCR4+ (GSE50821), VCAM+CD31-CD45-SCA1- or the YFP fraction from *Pax7$^{CreER/+}$;ROSA26$^{eYFP/+}$* cells (GSE47177). (B) Summary of the matrix illustrated in Fig. 1B. Genes with known function in myogenesis and muscle stem cells are indicated as examples in each cluster. Representative functional pathways are also indicated for each cluster. Analysis performed using GO and murine KEGG pathways.
