# Supplementary material for: Gene Expression Profiling of Muscle Stem Cells Identifies Novel Regulators of Postnatal Myogenesis
Source: Front Cell Dev Biol. 2016 Jun 21;4:58. doi: 10.3389/fcell.2016.00058 (PMC4914952; doi:10.3389/fcell.2016.00058)
Supplement: Supplementary file 13 [file Image4.PDF]

AlonsoMartinS_Supp fig4.

A Specific UR genes in the embryo

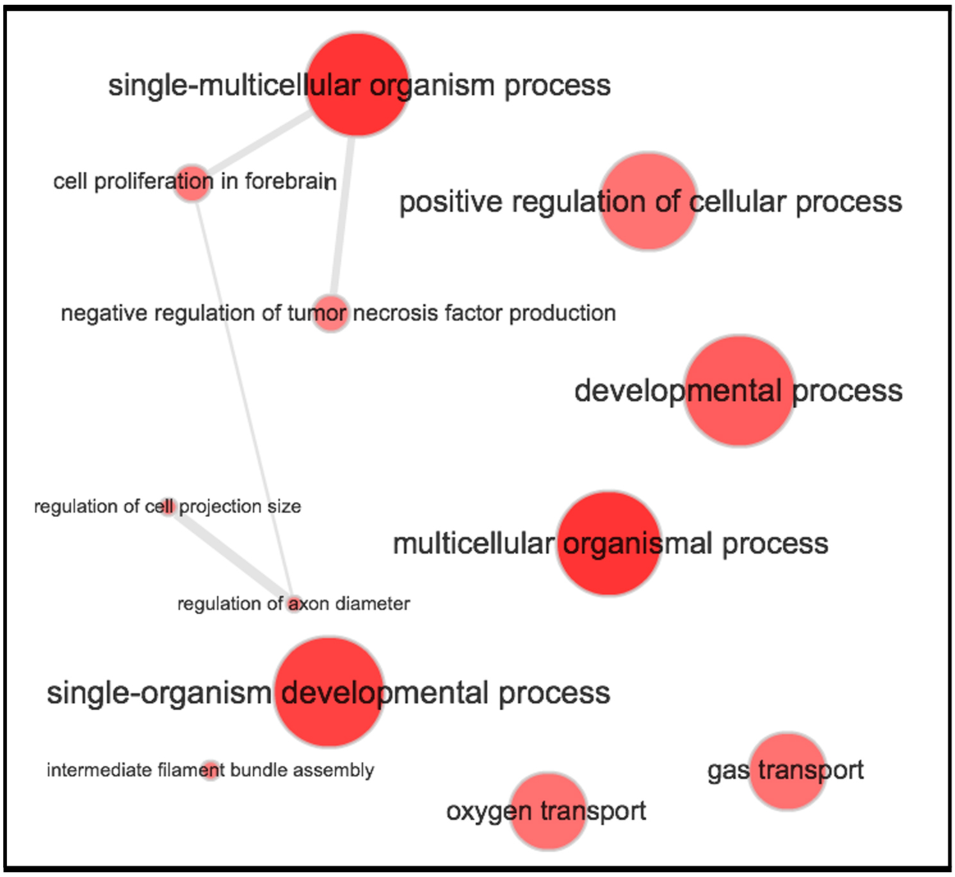

B Specific genes genes in fetal-early postnatal

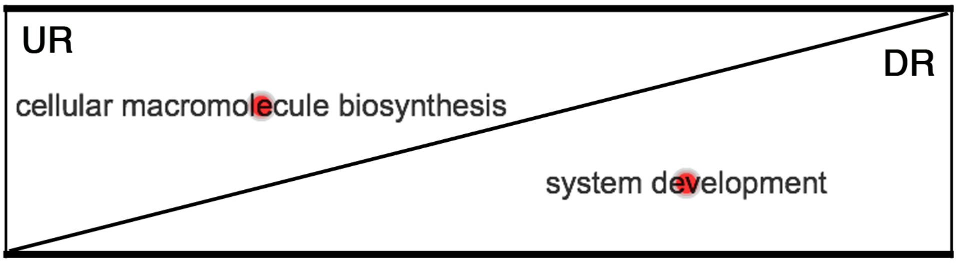

C Specific UR genes in the adult

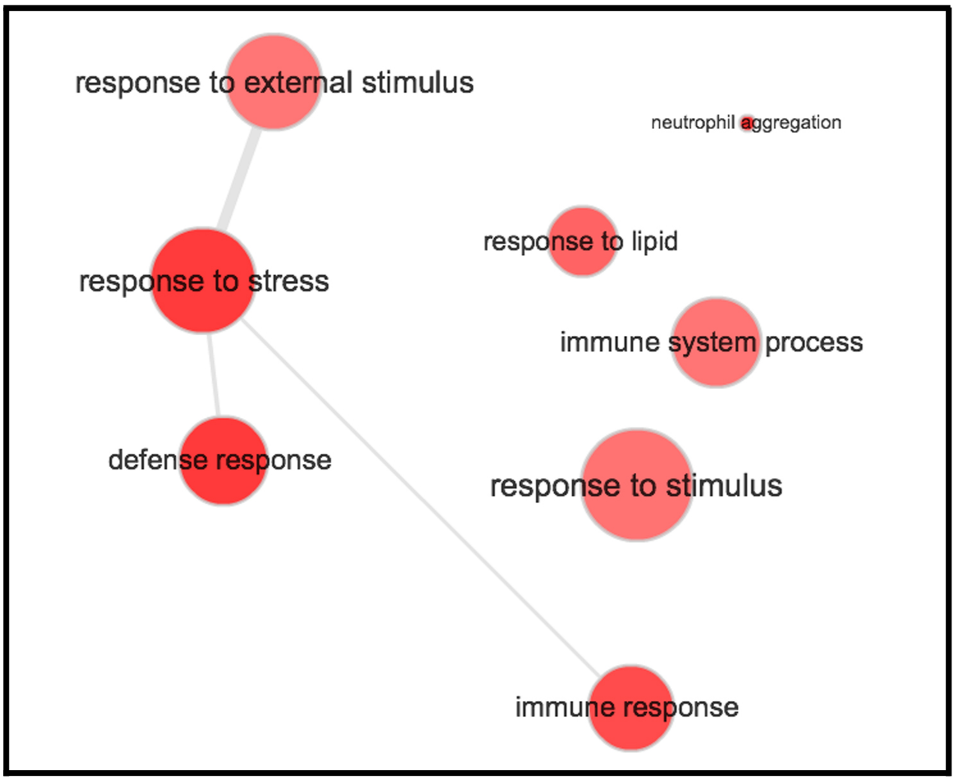

FIGURE S4: *In silico* analysis between muscle stem cell transitions, displaying most relevant pathways specific for each developmental signature. (A) Embryonic progenitors display 980 specific UR genes, grouped in 14 main GO processes (i.e. positive regulation of cellular and developmental processes or oxygen transport); and 631 specific DR genes included in 47 GO processes such as defence response, negative regulation of biological process, and response to stress. (B) Fetal-early postnatal progenitors show only 100 specific UR genes, included in one main GO process (cellular macromolecule biosynthesis), and 253 DR covering the "system development" GO process. (C) Quiescent satellite cells present 1005 specific UR genes, which represent nine GO processes, mostly related to stress and immune responses. In contrast, the 282 DR genes are represented in seven GO processes, related to signal transduction, regulation of multicellular organismal process and system development. See Fig. 1D.
Highly similar GO terms are linked by edges in the graph, where line width indicates the degree of similarity. Data obtained using REViGO software package.
