# Supplementary material for: Gene Expression Profiling of Muscle Stem Cells Identifies Novel Regulators of Postnatal Myogenesis
Source: Front Cell Dev Biol. 2016 Jun 21;4:58. doi: 10.3389/fcell.2016.00058 (PMC4914952; doi:10.3389/fcell.2016.00058)
Supplement: Supplementary file 14 [file Image5.PDF]

AlonsoMartinS_Supp fig5.

A

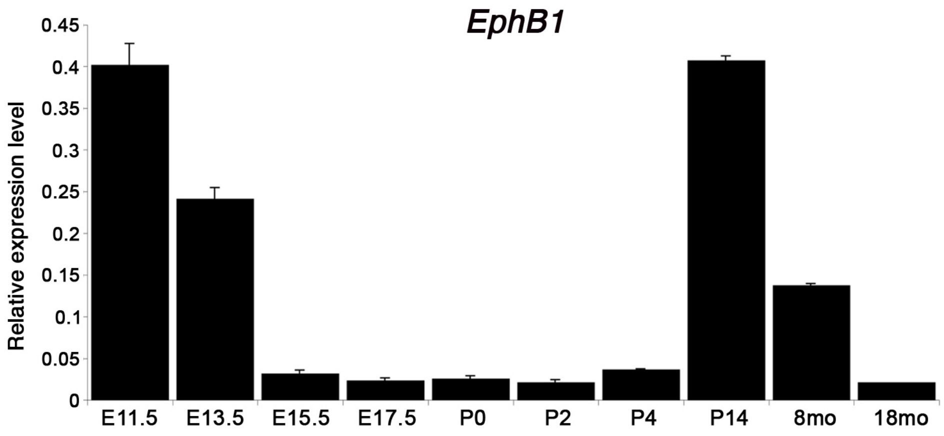

B

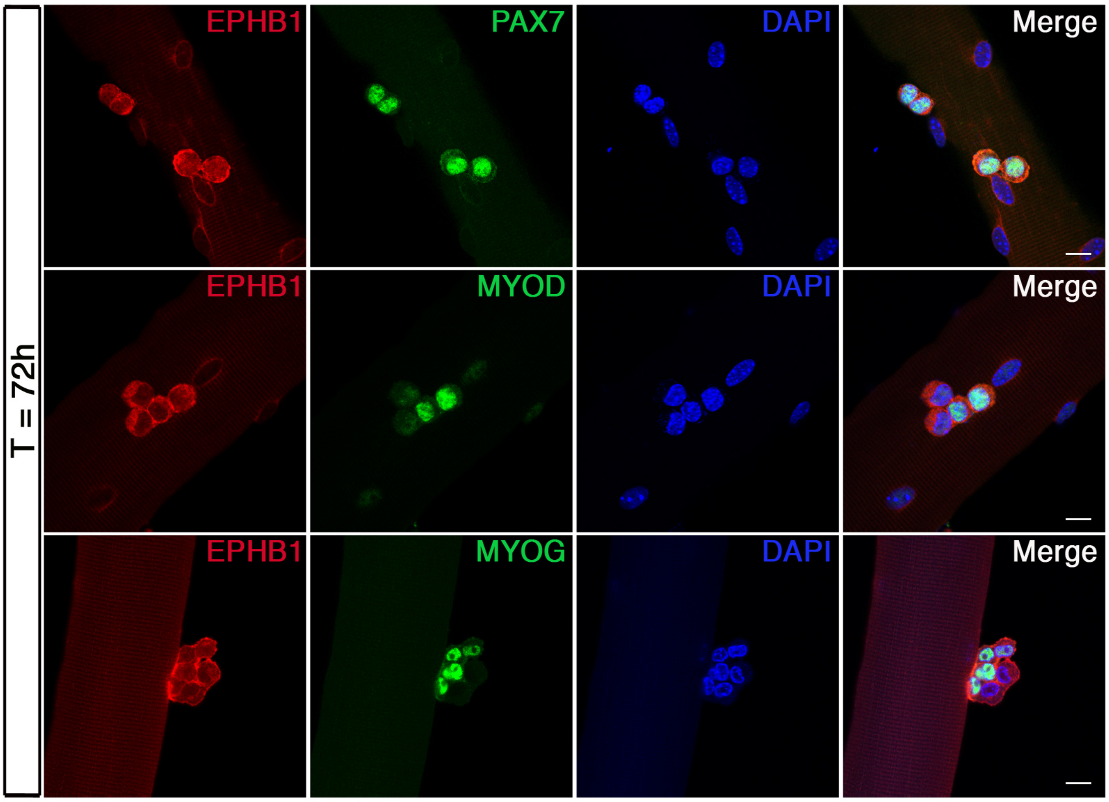

FIGURE S5: Validation of EPHB1 receptor analysis. (A) Quantitative RT-PCR *EphB1* expression is presented as shown (E11.5, E13.5, E15.5, E17.5, P0, P2, P4, P14, 8mo and 18 mo old) in FACS-sorted Pax3-GFP+ cells. These results confirm the data generated with the microarray. E, Embryonic days; P, Postnatal days; mo, age in months. (B) Expression of EPHB1 receptor in activated and proliferating satellite cells on isolated and cultured myofibers at T=72h. Immunostaining is shown for PAX7 (quiescence), MYOD (activation/proliferation) and MYOG (myogenin; differentiation) in green, and EPHB1 in red. Nuclei are labeled with DAPI (blue). Scale bars, 10µm.
