# Supplementary material for: Gene Expression Profiling of Muscle Stem Cells Identifies Novel Regulators of Postnatal Myogenesis
Source: Front Cell Dev Biol. 2016 Jun 21;4:58. doi: 10.3389/fcell.2016.00058 (PMC4914952; doi:10.3389/fcell.2016.00058)
Supplement: Supplementary file 15 [file Image6.PDF]

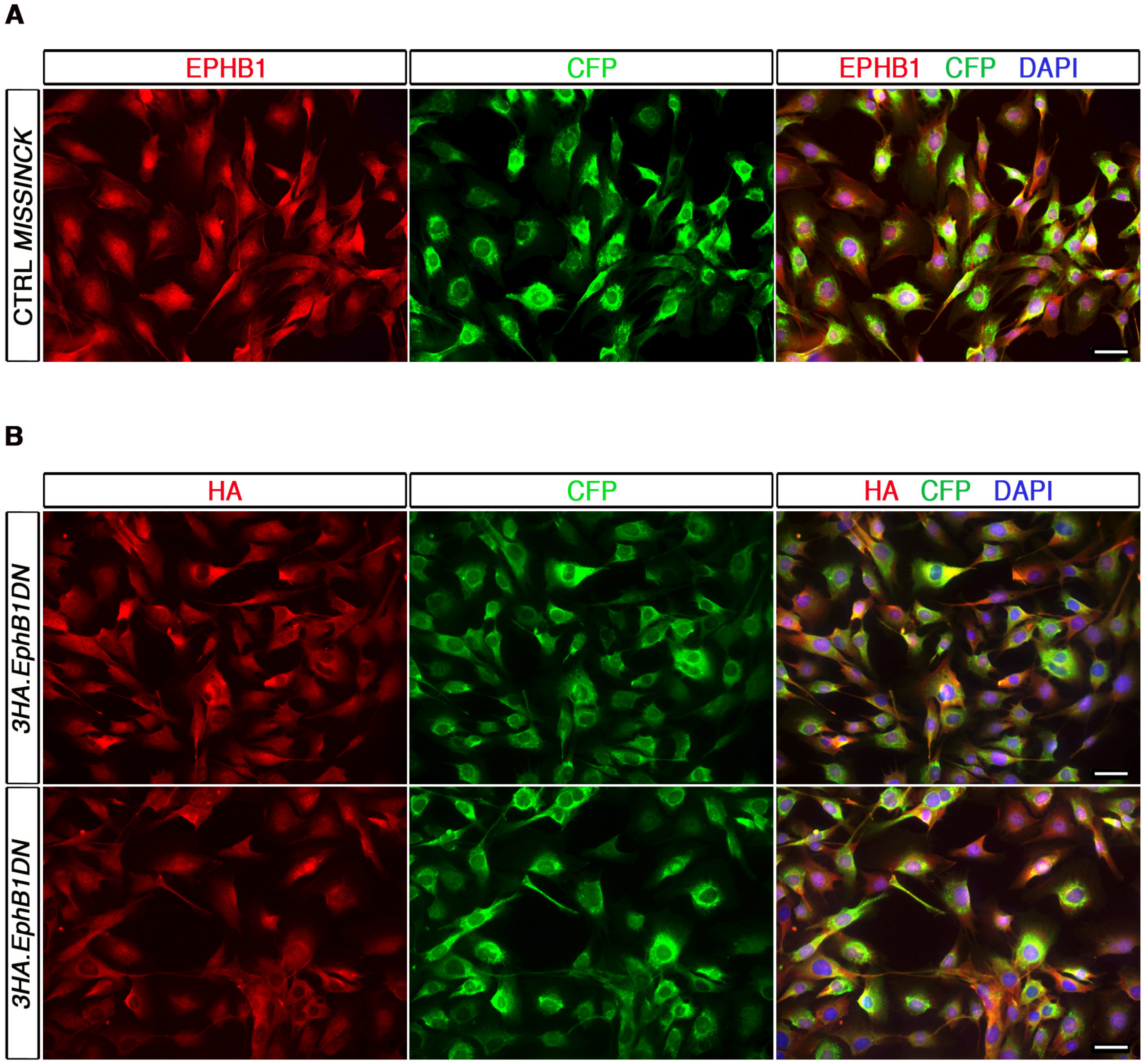

FIGURE S6: Infection of C2C12 with *MISSINCK* retroviral constructs. (A) *MIGR*-modified retroviral vector overexpressing Cyan Fluorescent Protein (CFP) in the endoplasmic reticulum and Golgi (*MISSINCK*) was used to report infected C2C12 cells (green). C2C12 cells express the EPHB1 receptor (red). (B) A HA-tagged *EphB1DN* was cloned in *MISSINCK* to localize the modified EPHB1 receptor in C2C12 cells, 48h after infection. Infected cells are shown in green (CFP), and HA antibody was used in red. Nuclei were counterstained with DAPI (blue). Scale bars, 25µm.
