# Supplementary material for: Gene Expression Profiling of Muscle Stem Cells Identifies Novel Regulators of Postnatal Myogenesis
Source: Front Cell Dev Biol. 2016 Jun 21;4:58. doi: 10.3389/fcell.2016.00058 (PMC4914952; doi:10.3389/fcell.2016.00058)
Supplement: Supplementary file 16 [file Image7.PDF]

AlonsoMartinS_Supp fig7.

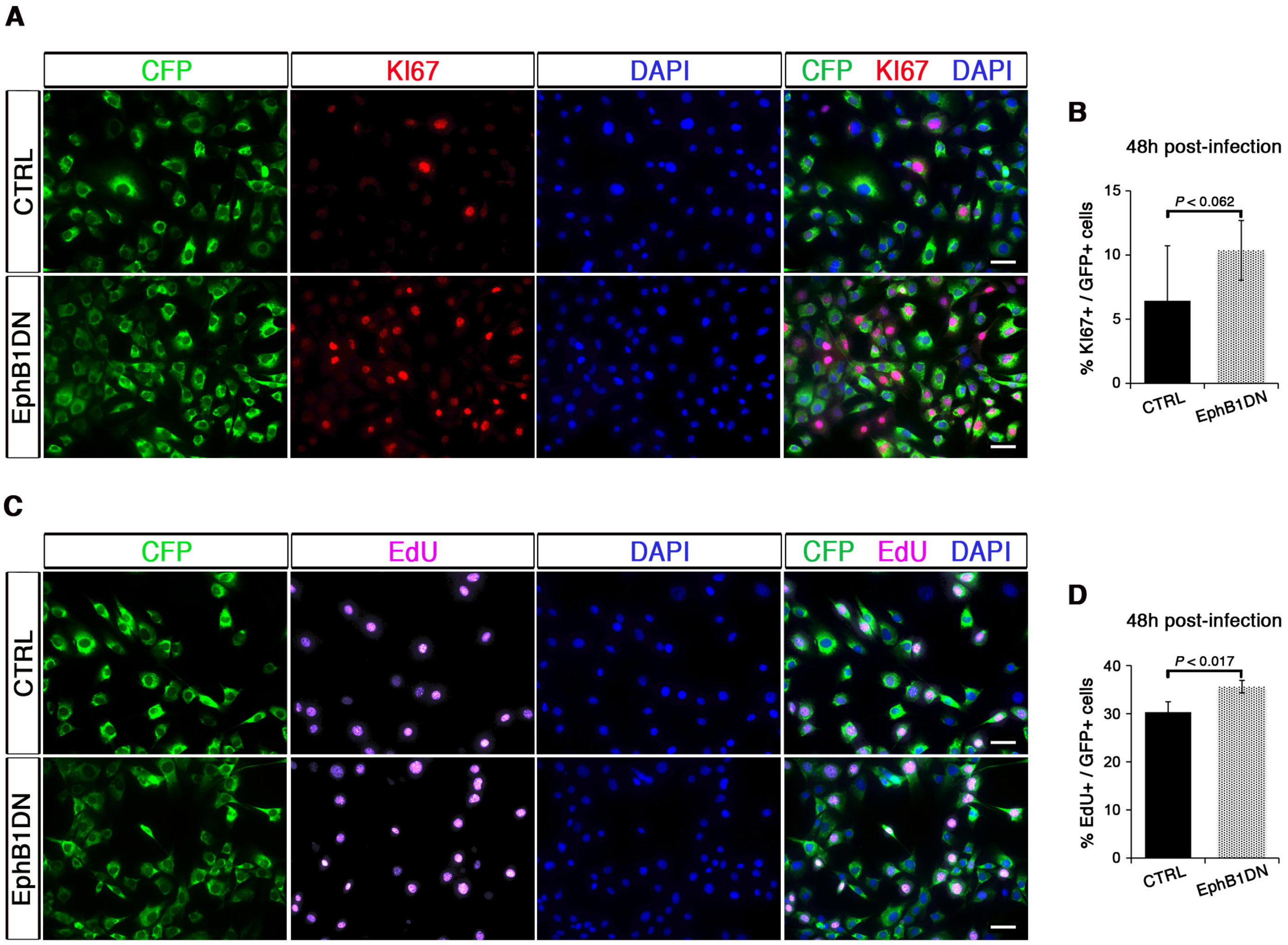

FIGURE S7: Infection of C2C12 with *MISSINCK-EphB1DN* retroviral constructs. (A) Immunofluorescence and (B) quantification of the proliferation marker KI67 (red) in infected C2C12 cells (CFP; green). Overexpressing a dominant negative EPHB1 receptor (EphB1DN) tends to increase the proliferation rate. (C) Immunofluorescence and (D) quantification of the proliferation marker EdU (magenta) in infected C2C12 cells (CFP; green). As before, overexpression of EphB1DN is translated into an increase in proliferation. This figure is related to Fig. 4A-B. Nuclei were counterstained with DAPI (blue). Scale bars, 25µm.
