# Supplementary material for: Gene Expression Profiling of Muscle Stem Cells Identifies Novel Regulators of Postnatal Myogenesis
Source: Front Cell Dev Biol. 2016 Jun 21;4:58. doi: 10.3389/fcell.2016.00058 (PMC4914952; doi:10.3389/fcell.2016.00058)
Supplement: Supplementary file 17 [file Image8.PDF]

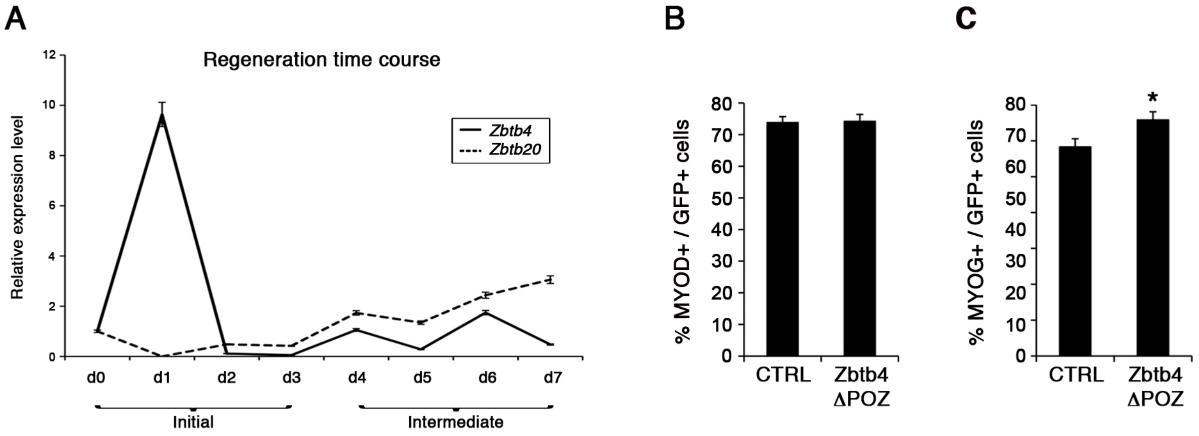

FIGURE S8: Expression profile of *Zbtb* factors during cardiotoxin-induced regeneration. (A) Total RNA was extracted on days 0-7, covering initial and intermediate steps of muscle regeneration, after cardiotoxin injection, in adult wild type *Tibialis anterior* muscles. (B-C) Quantifications for activation (MYOD; B) and differentiation (MYOG; C) are shown during overexpression of a dominant negative ZBTB4 (Zbtb4ΔPOZ) factor. The analysis was performed 48h after infection. *p*-value * $p<0.05$. Minimum number of infected satellite cells >200, for each marker analysis.
